# Integrative multi-omics analysis unveils stemness-associated molecular subtypes in prostate cancer and pan-cancer: prognostic and therapeutic significance

**DOI:** 10.1186/s12967-023-04683-6

**Published:** 2023-11-07

**Authors:** Kun Zheng, Youlong Hai, Yue Xi, Yukun Zhang, Zheqi Liu, Wantao Chen, Xiaoyong Hu, Xin Zou, Jie Hao

**Affiliations:** 1 Department of Urology, Shanghai Sixth People’s Hospital Affiliated to Shanghai Jiao Tong University School of Medicine, Shanghai, 200233 China; 2 Department of Reproductive Medicine, Central Hospital Affiliated to Shandong First Medical University, Jinan, 250013 Shandong China; 3 Beijing University of Chinese Medicine East Hospital, Zaozhuang Hospital, Zaozhuang, 277000 Shandong China; 4 Department of Oral and Maxillofacial Surgery, Zhongshan Hospital, Fudan University, Shanghai, 200032 China; 5 Shanghai Key Laboratory of Stomatology & Shanghai Research Institute of Stomatology, National Clinical Research Center of Stomatology, Ninth People’s Hospital, Shanghai Jiao Tong University School of Medicine, Shanghai, 200011 China; 6 Jinshan Hospital Center for Tumor Diagnosis & Therapy, Jinshan Hospital, Fudan University, Shanghai, 201508 China; 7 Department of Pathology, Jinshan Hospital, Fudan University, Shanghai, 201508 China; 8 Institute of Clinical Science, Zhongshan Hospital, Fudan University, Shanghai, 200032 China

**Keywords:** Prostate cancer, Stemness subtype, RNA sequencing, Pan‑cancer, Machine learning, Immunotherapy

## Abstract

**Background:**

Prostate cancer (PCA) is the fifth leading cause of cancer-related deaths worldwide, with limited treatment options in the advanced stages. The immunosuppressive tumor microenvironment (TME) of PCA results in lower sensitivity to immunotherapy. Although molecular subtyping is expected to offer important clues for precision treatment of PCA, there is currently a shortage of dependable and effective molecular typing methods available for clinical practice. Therefore, we aim to propose a novel stemness-based classification approach to guide personalized clinical treatments, including immunotherapy.

**Methods:**

An integrative multi-omics analysis of PCA was performed to evaluate stemness-level heterogeneities. Unsupervised hierarchical clustering was used to classify PCAs based on stemness signature genes. To make stemness-based patient classification more clinically applicable, a stemness subtype predictor was jointly developed by using four PCA datasets and 76 machine learning algorithms.

**Results:**

We identified stemness signatures of PCA comprising 18 signaling pathways, by which we classified PCA samples into three stemness subtypes via unsupervised hierarchical clustering: low stemness (LS), medium stemness (MS), and high stemness (HS) subtypes. HS patients are sensitive to androgen deprivation therapy, taxanes, and immunotherapy and have the highest stemness, malignancy, tumor mutation load (TMB) levels, worst prognosis, and immunosuppression. LS patients are sensitive to platinum-based chemotherapy but resistant to immunotherapy and have the lowest stemness, malignancy, and TMB levels, best prognosis, and the highest immune infiltration. MS patients represent an intermediate status of stemness, malignancy, and TMB levels with a moderate prognosis. We further demonstrated that these three stemness subtypes are conserved across pan-tumor. Additionally, the 9-gene stemness subtype predictor we developed has a comparable capability to 18 signaling pathways to make tumor diagnosis and to predict tumor recurrence, metastasis, progression, prognosis, and efficacy of different treatments.

**Conclusions:**

The three stemness subtypes we identified have the potential to be a powerful tool for clinical tumor molecular classification in PCA and pan-cancer, and to guide the selection of immunotherapy or other sensitive treatments for tumor patients.

**Supplementary Information:**

The online version contains supplementary material available at 10.1186/s12967-023-04683-6.

## Introduction

Cancer poses a significant threat to human health and imposes a substantial burden on the global public health. It is the leading cause of death worldwide [1, 2]. Prostate cancer (PCA) is one of the most common malignant tumors in men, and its incidence has increased significantly in recent years [3, 4]. Despite progress in its treatment, such as surgery, radiotherapy, and chemotherapy, the disease remains a challenge, particularly in cases of castrate-resistant prostate cancer [5, 6]. Immunotherapy, which has achieved excellent therapeutic effects in various tumors, is a revolutionary breakthrough in tumor treatment [7, 8]. However, immune checkpoint blockade therapy (ICB) has limited efficacy in unselected patients with PCA, and only a small subgroup of patients may be sensitive to ICB [9]. Therefore, determining patient subtypes that can benefit from immunotherapy is an urgent problem to be solved. Several large-scale clinical trials are currently ongoing, such as the phase III clinical trials of pembrolizumab (KEYNOTE-991) and nivolumab plus docetaxel (Checkmate 7DX), to explore the benefits of immunotherapy with/without other conventional treatments for PCA patients [10]. In recent years, numerous biomarkers that can affect ICB efficiency have been discovered, such as PD-L1, microsatellite instability, tumor mutational burden (TMB), and TCR polymorphism [11]. However, they are far from being ideal biomarkers for PCA [10].

The molecular subtyping of tumors can be used to guide the precision diagnosis and treatment of tumor patients. Cellular and molecular characteristics of tumors have shown potential in precision therapy of PCA, such as cancer-associated fibroblasts (CAFs) and their signature genes [12, 13]. Serum prostate-specific antigen (PSA) is the most important marker for PCA; however, it has been criticized for its poor specificity [14]. Existing molecular subtyping methods for PCA were designed for specific clinical applications. For example, the PAM50 method is used to guide androgen deprivation therapy [15]; the Decipher method is used to guide radiotherapy and surgical treatment [16]. In addition, existing biomarkers have limited clinical utility and are not suitable for guiding ICB treatment [17–19]. There is still lacking subtyping methods for PCA, which can effectively and systematically characterize patients from various clinical points of view, e.g., diagnosis, prognosis, recurrence risk, metastasis risk, progression risk, and efficacies of different treatments.

Stemness refers to the self-renewal and differentiation potential of cells. In almost all human malignant tumors, there is a rare subset of cancer cells with stem-like properties, called “cancer stem/stem-like cells” (CSCs) [20]. CSCs are considered the origin cells of tumors and play an important role in the recurrence, metastasis, and treatment resistance of many tumors, including PCA [21–23]. They also affect the effectiveness of immunotherapy [24]. Therefore, stemness based subtyping holds potential in personalized management of PCA patients.

In this study, we utilized single-cell RNA-seq (scRNA-seq), bulk RNA-seq, methylation array, and whole exon sequencing datasets to systematically assess stemness differences among PCA patients. By integrating scRNA-seq and bulk RNA-seq, we developed a stemness-based subtyping model consisting of 18 stemness related gene-sets that separated PCA samples into three subtypes with distinctive clinical and molecular characteristics, functional annotations, prognoses, and treatment responses, especially immunotherapy. Furthermore, a subtype predictor including 9 stemness related genes was constructed which showed great performance in tumor diagnosis, predicting ICB and androgen deprivation therapy (ADT) response, metastasis, recurrence, progression, and prognosis.

## Results

### Stemness scores are closely correlated with clinical and molecular features

#### Single-cell and bulk RNA-seq analyses show a positive correlation between stemness and PCA malignancy

**Fig. 1:**
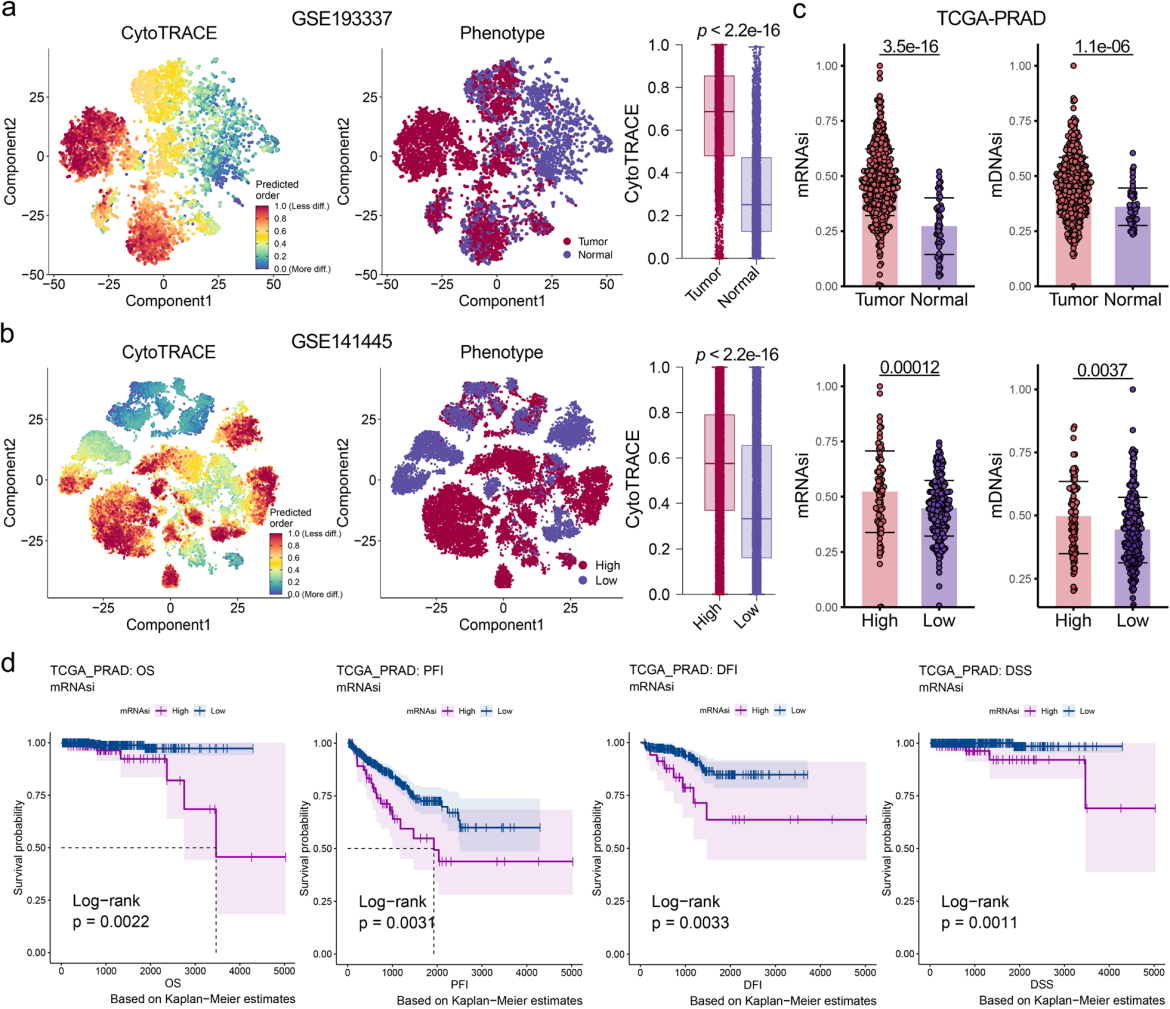
Correlation of stemness levels with clinical, pathological, and molecular features in patients with prostate cancer (PCA).** a** t-distributed stochastic neighbor embedding (t-SNE) plot of malignant and benign epithelial cells from GSE193337 dataset (medium), along with their corresponding stemness scores (cytoTRACE, left), and the comparison of these scores between two groups (right). **b** t-SNE plot of high and low grade PCA cells from GSE141445 dataset (medium), along with their corresponding cytoTRACE scores (left), and the comparison of these scores between two groups (right). **c** Comparison of stemness scores (mRNAsi, mDNAsi) between benign and malignant prostate samples, as well as between high (Gleason score [GS] > 7) and low (GS < 7) grade PCA samples from TCGA-PRAD. **d** Kaplan–Meier (K–M) analysis demonstrated a correlation between the mRNAsi scores and the prognosis of PCA patients from TCGA-PRAD. *OS* overall survival, *PFI* progression-free interval, *DFI* disease-free interval, *DSS* disease-specific survival. Dashed line: median survival time. Color range: 95% confidence interval (CI)

The workflow of this study is depicted in Additional file 1: Fig. S1 and Methods S1, and all the datasets used in this study are presented in Additional file 1: Table S1. Prostate epithelial cells were extracted from five PCA scRNA-seq datasets [25–29] (Additional file 1: Fig. S2) and the cytoTRACE algorithm [30] was used to calculate their stemness levels. Consistently, the results showed significantly higher cytoTRACE scores in the malignant epithelium than in the para-cancerous or benign prostate epithelium (Fig. 1a, Additional file 1: Figs. S3a–c). Moreover, among malignant epithelial cells, high-grade PCA (Gleason score [GS] > 7) showed significantly higher stemness scores than low-grade PCA (GS ≤ 7) (Fig. 1b, Additional file 1: Fig. S3d).

In parallel, there are stemness indices calculated by the one-class logistic regression (OCLR) algorithm based on specific stemness probes, including mRNAsi obtained from bulk RNA-seq data and mDNAsi, EREG_mDNAsi, and DMPsi obtained from DNA methylation data [31]. We computed the stemness indices of 553 patients from The Cancer Genome Atlas-Prostate Adenocarcinoma (TCGA-PRAD). These results further confirmed the findings obtained from the scRNA-seq (Fig. 1c, Additional file 1: Fig. S3e). Likewise, these results were consistently replicated in six other PCA bulk RNA-seq datasets [32–36] (Additional file 1: Fig. S3f).

#### Stemness scores are significantly associated with PCA clinicopathological and molecular features

We divided the patients into two groups based on their stemness indices. Kaplan–Meier (K–M) analysis showed that the high stemness indices group had remarkably poorer overall survival (OS), progression-free interval (PFI), disease-free interval (DFI), and disease-specific survival (DSS) (Fig. 1d, Additional file 1: Fig. S3g–i). Furthermore, we demonstrated the association between stemness and worse prognosis on another six PCA datasets [32, 33, 37–40] (Additional file 1: Fig. S3j). Based on these results, we conclude that stemness is a risk factor for the prognosis of PCA.

Additionally, we found that mRNAsi is closely related with serum PSA, androgen receptor (AR), tumor purity, pathological T stage (pT), as well as somatic copy number alterations (SCNAs) and TMB (Additional file 1: Fig. S3k, l, Remark A). Furthermore, we observed a negative correlation between stemness and immune infiltration levels (Additional file 1: Fig. S4, Remark B).

### Identification of three PCA subtypes based on stemness and their association with prognosis

#### Development of 18 stemness signatures for subtyping

**Fig. 2:**
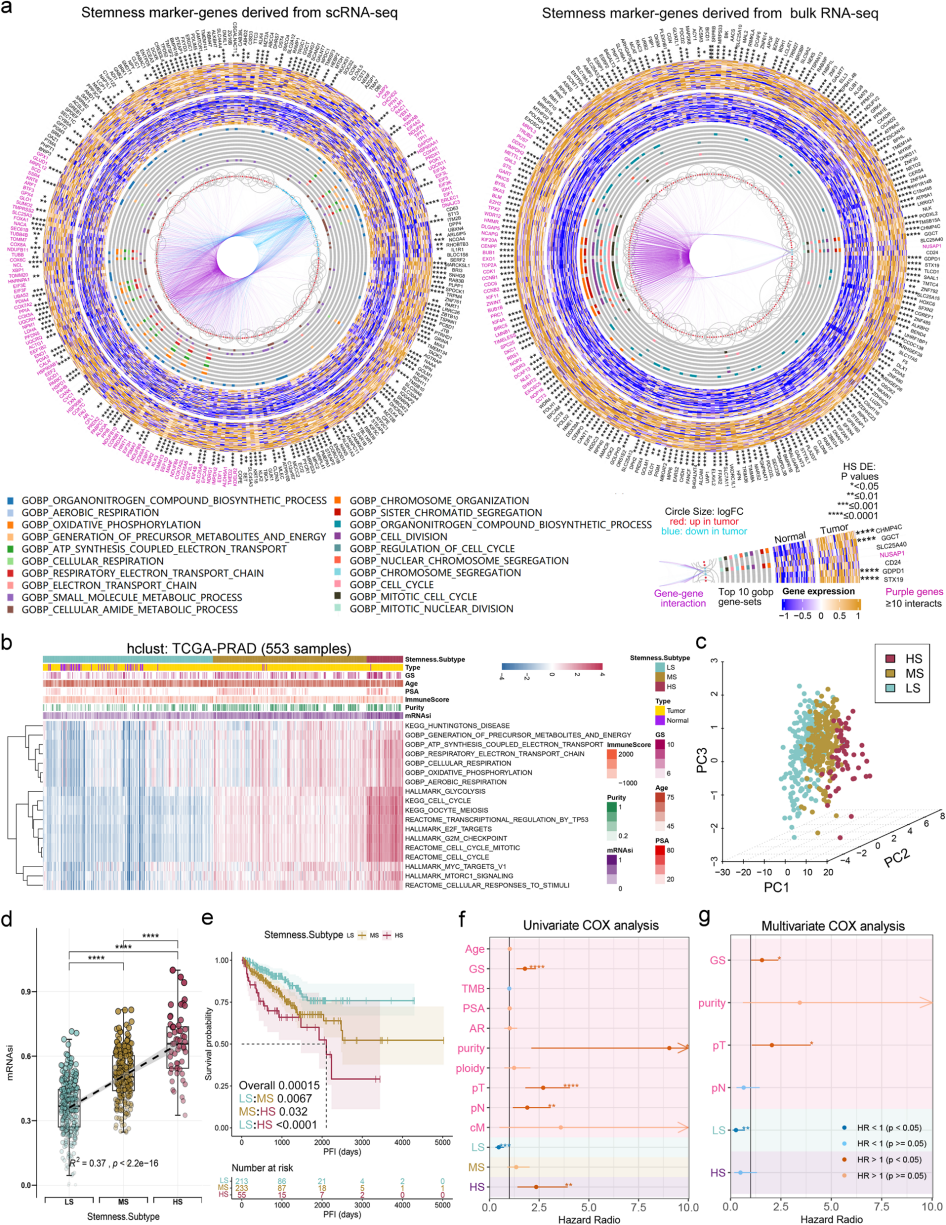
Identification of three PCA stemness subtypes based on stemness signatures.** a** CircosPlot shows 288 stemness marker genes obtained from scRNA-seq data that are significantly positively correlated with cytoTRACE and significantly upregulated in both malignant and high-grade PCA cells (left), and 220 stemness marker genes derived from bulk RNA-seq data that are significantly positively correlated with mRNAsi and significantly upregulated in PCA samples (right). **b** Unsupervised hierarchical clustering based on the activity scores of the 18 stemness signatures classified PCA patients from TCGA-PRAD into three subtypes: low stemness (LS), medium stemness (MS), and high stemness (HS) subtypes. **c** 3D projection of the principal components obtained through PCA analysis. **d** Levels and trends of stemness score (mRNAsi) within three stemness subtypes. **e** Three stemness subtypes of TCGA-PRAD exhibits distinct PFI outcomes. **f**, **g** Univariate (**f**) and multivariate (**g**) Cox regression analysis of the three stemness subtypes, and clinical and molecular characteristics. *p < 0.05, **p < 0.01, ***P < 0.001, ****p < 0.0001

Since there was a significant correlation between stemness scores and PCA prognosis, we postulated that certain signatures indicating the tumor’s stemness status could be utilized for patient classification. Therefore, we conducted a joint analysis of five scRNA-seq [25–29] (75,350 cells) and seven bulk RNA-seq datasets [32–36] (1641 samples) to develop these signatures. The development process was illustrated in Additional file 1: Fig. S5a. We identified 288 genes from the scRNA-seq data that showed a significant positive correlation with cytoTRACE scores and were significantly overexpressed in both tumors (compared to normal samples) and high-grade PCAs (compared to low-grade PCAs, Fig. 2a, left). Additionally, we identified 220 genes from the bulk RNA-seq data that exhibited a significant positive correlation with mRNAsi and were highly expressed in the tumor samples (Fig. 2a, right). After applying union, gene set enrichment analysis (GSEA), and univariate COX analysis, we identified 18 gene-sets representing risk factors (Additional file 1: Fig. S5b) for subsequent stemness subtyping, including 160 genes. Among these 18 gene sets, 7 were metabolic-related, 8 were cell cycle-related, and the other 3 were MYC targets, mTORC1 signaling, and cellular responses to stimuli, all of which are classic oncogenic signaling pathways [41, 42] (Additional file 1: Fig.S5c, Additional file 2: Data S1).

#### Identification of three stemness subtypes

Subsequently, based on the ssGSEA scores of these 18 signatures, we used unsupervised hierarchical clustering to classify the 553 samples from TCGA-PRAD into three subtypes (Fig. 2b, c, Additional file 1: Fig. S6a). We defined the group with the highest scores, which contained 56 samples (10.1%), as the “High Stemness” (HS) subtype, while the group with the lowest scores, consisting of 261 samples (47.2%), was named the “Low Stemness” (LS) subtype. The medium score group, comprising 236 samples (42.7%), was referred to as the “Medium Stemness” (MS) subtype, indicating that they were in a transitional state with the potential to transform into either a high or low stemness status. To validate our classification results, we compared the four aforementioned stemness indices across the three subtypes. Compared to LS, mRNAsi, mDNAsi, EREG_mDNAsi, DMPsi scores, and tumor purity were all significantly higher in HS and intermediate in MS (Fig. 2d, Additional file 1: Fig. S6b).

#### Significant differences in prognosis among patients with three stemness subtypes of PCA

K–M analysis revealed that HS patients had the worst PFI, whereas LS patients had the longest PFI (Fig. 2e, p = 0.00015), indicating that HS patients may experience disease progression earlier after treatment. However, there were no significant differences in OS, DFI, or DSS among the three subtypes (Additional file 1: Fig. S6c). Univariate COX analysis showed that LS and HS were protective factors and risk factors for PFI, respectively (Fig. 2f). Moreover, the multivariate COX analysis further demonstrated that LS was an independent protective factor for PFI (Fig. 2g).

Finally, we further validated the stability and universality of the stemness classification method by using two additional algorithms, non-negative matrix factorization (NMF) [43] and CensusClusterPlus [44], along with seven independent PCA bulk RNA-seq datasets [32, 33, 37–40, 45] (Additional file 1: Fig. S6d–u, Remark C).

### Three stemness subtypes have distinct clinical features, mutational events, and functional annotations

#### Significant differences in certain clinicopathological features

**Fig. 3:**
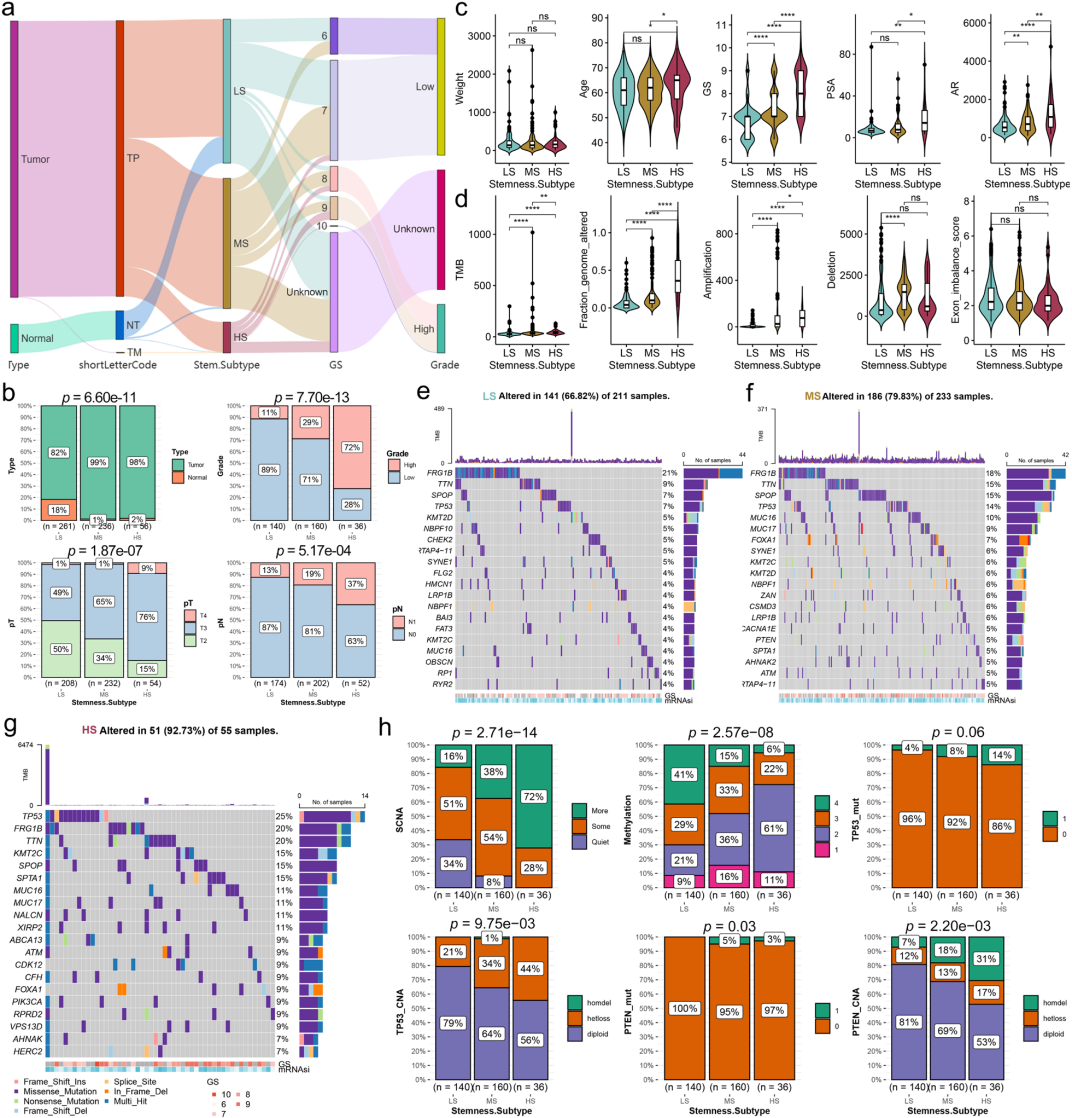
Comparison of clinicopathological and molecular features among three PCA stemness subtypes.** a** Sankey diagram showing sample flow for stemness subtype, sample type, and GS. **b** Comparison of sample type, grade, pT and pN among the three stemness subtypes. **c** Comparison of patient weight, age at diagnosis, GS, PSA, and AR among three stemness subtypes. **d** Comparison of TMB, fraction genome altered, amplifications, deletions, and exon imbalance scores among three stemness subtypes. **e**–**g** Oncoplots showing the top 10 mutated genes in LS (**e**), MS (**f**) and HS (**g**). **h** Stacked histograms showing comparisons of somatic copy number alterations (SCNAs), DNA methylation clustering, TP53 mutations and CNAs, and PTEN mutations and CNAs among the three stemness subtypes. *p < 0.05, **p < 0.01, ***P < 0.001, ****p < 0.0001

Next, we compared the demographic and clinicopathological characteristics of the three stemness subtypes in patients with PCA. We found that LS had the highest proportion of para-cancerous samples (Fig. 3a, b; LS:MS:HS = 18%:1%:2%, p = 6.6e−11). The proportion of high-grade PCAs (GS > 7), pT3 and pT4 PCAs, and lymph node metastasis (N1) patients gradually increased in the LS, MS, and HS subtypes (Fig. 3a and b). There were no significant differences in body weights among the three subtypes (Fig. 3c). Compared with LS and MS, HS patients were older at diagnosis (Fig. 3c), suggesting that PCA patients diagnosed at an older age may have a relatively higher stemness status. Moreover, Gleason score, PSA, and androgen receptor were all significantly higher in HS patients (Fig. 3c), indicating that HS patients with the highest malignancy may benefit more from ADT treatment.

#### Significant differences in mutation events

Genome changes are research hotspots; therefore, we performed somatic mutation analysis. TMB, fragment genomic alteration (FGA), and amplification were all significantly higher in the HS subtype (Fig. 3d), suggesting that the HS subtype may be more likely to respond to immunotherapy [46]. The deletion was significantly higher in the MS subtype than in the LS subtype (Fig. 3d). Exon imbalance scores did not differ between stemness subtypes (Fig. 3d). Oncoplot revealed that the HS subtype had the highest rate of genomic alterations (Fig. 3e–g, LS:MS:HS = 66.82%:79.83%:92.73%). Although the top mutated genes of the three subtypes are similar, there are great differences in their mutation rates (Fig. 3e–g). We also observed that TP53 and ABCA13 mutations co-occurred, as did TTN and HERC2 mutations in the HS subgroup (Additional file 1: Fig. S7a). Silencing of TP53, a tumor suppressor gene, and inactivation of ABCA13, a cholesterol transporter protein, may be possible mechanisms contributing to the highly malignant phenotype of HS [47, 48]. In the LS subtype, we found a co-mutation of TTN and FAT3 (Additional file 1: Fig. S7b), while SPOP and TP53 mutations were mutually exclusive in the MS subtype (Additional file 1: Fig. S7c). The proportion of more SCNAs cluster gradually increased in the stemness subtypes (Fig. 3h, LS:MS:HS = 16%:38%:72%, p = 2.7e−14), while the high methylation cluster decreased successively (Fig. 3h, p = 2.6e−8). We also investigated the status of mutations and copy number alterations (CNAs) in common biomarkers of PCA. We found that the proportion of TP53 mutations and CNAs (heterozygously deleted [hetloss] + homozygously deleted [homdel]) increased progressively in the three subtypes (Fig. 3h, p = 0.06 and 0.0098, respectively). Compared with LS, the proportion of PTEN mutations was significantly higher in MS and HS (p = 0.03), and the proportion of CNAs increased in these three subtypes (Fig. 3h, p = 0.0022). Similarly, both the mutations and CNAs of CDKN1B and CNAs of RAD51C, FAM175A, CHD1, RB1, FANCC, and SPOPL were significantly higher in HS than in LS (Additional file 1: Fig. S7d). However, we found no significant differences in the mutations and CNAs of BRCA1 and BRCA2 between the three subtypes (Additional file 1: Fig. S7d). Understanding the mutation landscape of the aforementioned stemness subtypes is beneficial for uncovering potential mechanisms underlying tumor development, and provides a basis for discovering potential therapeutic targets and biomarkers.

#### HS enriches oncogenic signaling pathways

Subsequently, we performed gene set variation analysis [49] (GSVA), GSEA [50], and ingenuity pathway analysis (IPA) to investigate the functional annotations, signaling pathways, and underlying mechanisms associated with the PCA stemness subtypes. As shown in Additional file 1: Fig. S8, the LS subtype mainly enriched nonspecific pathways. In contrast, HS exhibited enrichment in cell cycle-related signaling pathways [51], but IPA showed that the activity of these signaling pathways was suppressed. MS-enriched signaling pathways were similar to those of HS, but with slightly lower expression levels. See Additional file 1: Remark D for more details.

### Three stemness subtypes have different treatment sensitivity and TIME patterns

#### Three stemness subtypes retain sensitivity to specific drugs

**Fig. 4:**
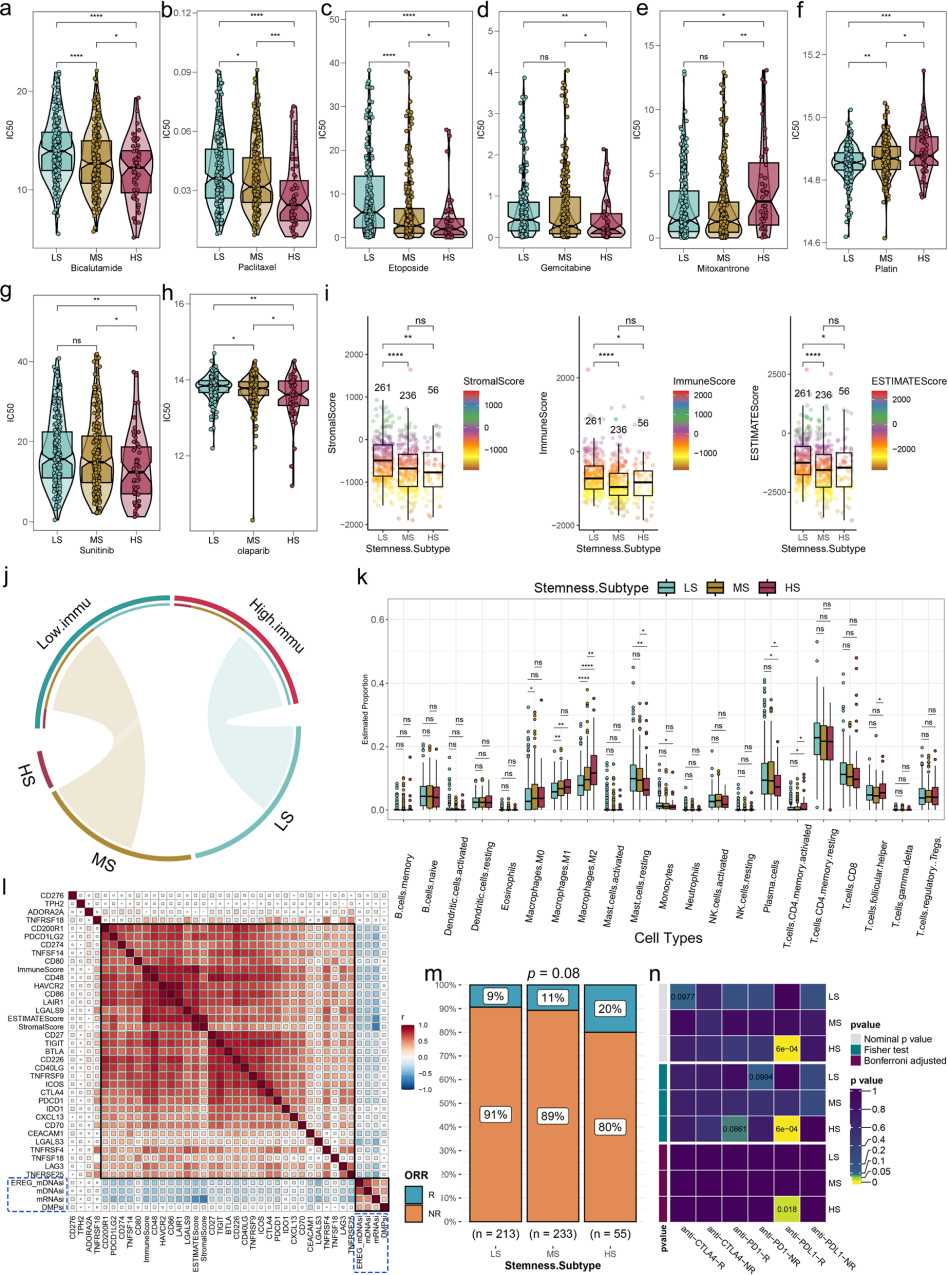
Comparison of drug sensitivities and TIME patterns among three PCA stem subtypes.** a**–**h** Comparisons of sensitivities of three stemness subtypes to clinically preferred and recommended drugs. **i** Differences in TME scores among the three stemness subtypes. **j** Hypergeometric tests reveal an association between stemness subtypes and TIME subtypes. **k** Boxplots showing comparisons of immunocyte abundance among the three stemness subtypes. **l** Correlation heatmap showing the correlation between stemness indices and expression levels of immune checkpoint molecules. **m** Stacked histogram showing differences in responsiveness of the three PCA stemness subtypes to immune-checkpoint blockade (ICB) therapy. Evaluated by TIDE algorithm. **n** Submap analysis reflects the sensitivity of the three PCA stemness subtypes to an-PD-1, anti-PD-L1 and anti-CTLA-4 treatments. *p < 0.05, **p < 0.01, ***P < 0.001, ****p < 0.0001

Given that the three stemness subtypes have unique functional pathways, we used the oncoPredict package [52] to predict the sensitivity of the three stemness subtypes to drugs and quantified sensitivity using half-maximal inhibitory concentrations (IC50). For the selection of conventional drugs, HS and MS subtypes were more sensitive to ADT (bicalutamide, abiraterone) (Fig. 4a, Additional file 1: Fig. S9a); for chemotherapy drugs, HS was more sensitive to taxanes (paclitaxel, docetaxel) (Fig. 4b, Additional file 1: Fig. S9b), etoposide (Fig. 4c), and gemcitabine (Fig. 4d), but resistant to mitoxantrone (Fig. 4e) and platinum drugs (Fig. 4f). In terms of recommended drug selection, HS demonstrated greater sensitivity to epidermal growth factor receptor (EGFR) inhibitors such as sunitinib (Fig. 4g), sorafenib, and imatinib (Additional file 1: Fig. S9c, d), while showing lower sensitivity to cabozantinib, afatinib, and erlotinib (Additional file 1: Fig. S9e–g). Additionally, HS showed increased sensitivity to poly (ADP-ribose) polymerase (PARP) inhibitors, including olaparib and talazoparib (Fig. 4h, Additional file 1: Fig. S9h), as well as to BI-2536 (Fig. S9j), histone deacetylase (HDAC) inhibitors (tacedinaline) (Additional file 1: Fig. S9k), and TGF-β receptor inhibitors (SB-431542) (Additional file 1: Fig. S9l). Conversely, MS exhibited greater sensitivity to anti-EGFR (cetuximab) (Additional file 1: Fig. S9m) and LS was more responsive to cyclin-dependent kinase (CDK) inhibitors (AZD5438) (Additional file 1: Fig. S9n).

#### Three stemness subtypes have distinct TIME patterns and immunotherapy responsiveness

As demonstrated above, the stemness scores exhibited an inverse correlation with immune infiltration in PCA (Additional file 1: Fig. S4). To further investigate the relationship between stemness subtypes and their response to immunotherapy, we examined the TIME patterns. Our results indicated that the LS subtype had the highest Stromal, Immune, and ESTIMATE scores compared to the MS and HS subtypes (Fig. 4i). This indicated that the LS subtype had the most abundant immune infiltration. Moreover, we observed a significant association between the LS subtype and the High.immu subtype, whereas the MS subtype was significantly associated with the Low.immu subtype (Fig. 4j, Additional file 1: Fig. S9o).

We utilized the CIBERSORT algorithm [53] to assess immunocyte infiltration levels in the 553 PCA samples. Our findings revealed that CD4T, M1 and M2 macrophages had the highest abundance, while mast and plasma cells showed the lowest infiltration in HS (Fig. 4k). Moreover, Spearman correlation analysis showed a dramatically negative correlation between the stemness indices and the majority of immune checkpoint molecules (Fig. 4l), and most molecules were least expressed in HS (Additional file 1: Fig. S9p). These differences in TIME patterns and immune checkpoint molecule expression could potentially affect the efficacy of immunotherapy. Therefore, we applied the TIDE algorithm [54] to estimate the response of the patients to immunotherapy. The results showed that the proportion of responders in the HS was higher than that in the LS and MS subtypes (Fig. 4m, Additional file 1: Fig. S9q, p = 0.08). Additionally, the submap analysis [55] revealed that HS was the most sensitive to anti-PD-L1 (Fig. 4n).

### Construction and validation of stemness subtype predictor

#### Construction of stemness subtype predictor

**Fig. 5:**
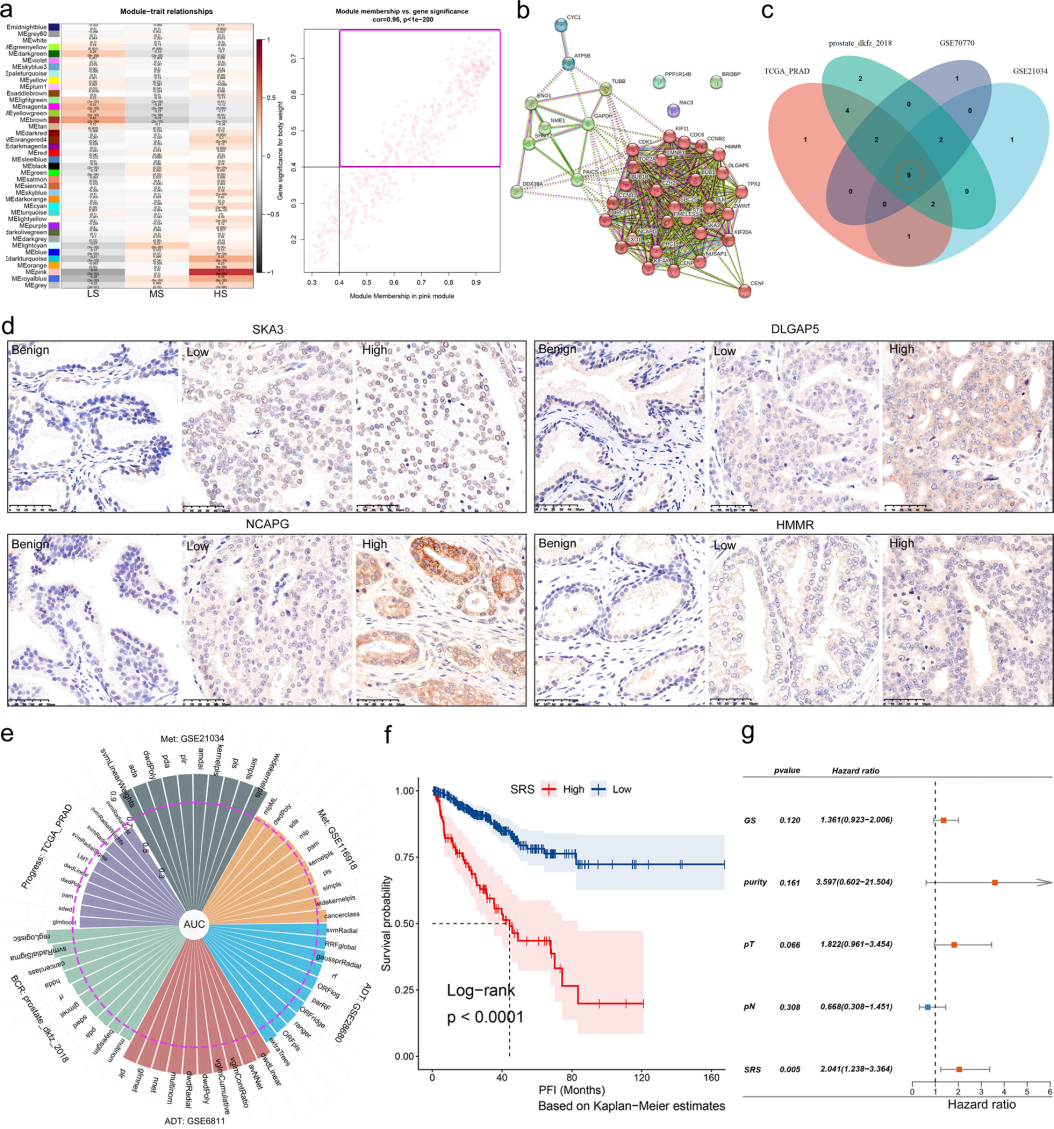
Construction and validation of stemness subtype predictor.** a** Correlation analysis between module eigengenes and stemness subtypes of TCGA-PRAD (left). The highest correlation between GS and MM in the pink module. Dots within the pink rectangle were defined as HS hub genes (right). **b** Protein–protein interaction (PPI) network of the 40 genes of Core.Sig, and these proteins were divided into three clusters based on the MCL inflation parameter. **c** Venn diagram identified the nine most critical stemness subtype marker genes that were intersected by 4 datasets and 76 machine learning algorithms (MLs). **d** Immunohistochemistry (IHC) staining shows the protein levels of four critical stemness subtype marker genes (SKA3, DLGAP5, NCAPG, HMMR) in benign, low-grade, and high grade PCA samples. Representative images are shown. **e** Histogram shows the performances of the 9-gene predictor in distinguishing benign from malignant tumors and predicting androgen deprivation therapy (ADT) response, metastasis, biochemical recurrence and progression via 100 MLs. The top 10 MLs with the best performance are exhibited. **f** K–M analysis shows the effect of 9-gene-based stemness-related risk score (SRS) on PFI of PCA patients from TCGA-PRAD. **g** Multivariate COX analysis showed that SRS was the most important independent risk factor for PCA patients

To facilitate the clinical application of our findings, we developed a stemness subtype predictor with high sensitivity and specificity, using the process outlined in Additional file 1: Fig. S10a (see Additional file 1: Methods S1 for details). We performed weighted gene co-expression network analysis (WGCNA) [56] (Fig. 5a, Additional file 1: Fig. S10b–g), protein–protein interaction (PPI) analysis (Fig. 5b), and Venn diagram plotting in sequence, and ultimately obtained 9 most critical stemness marker genes (Fig. 5c, Additional file 1: Table S2). We observed that these genes were significantly overexpressed in tumors (compared to normal, Additional file 1: Fig. S10h) and high-grade PCAs (compared to low-grade, Additional file 1: Fig. S10i).

We conducted a literature review on the research status of genes in PCA. CDK1 [57], KIF4A [58], TPX2 [59], BUB1 [60], and TOP2A [61] have been reported to promote PCA progression. However, there is a lack of evidence regarding the roles of SKA3, DLGAP5, NCAPG, and HMMR in PCAs. We collected 60 PCA samples and performed immunohistochemistry (IHC) to verify the expression of these four proteins. Our findings showed that their expression gradually increased in benign prostate tissues, and in low-grade and high-grade PCAs (Fig. 5d).

#### Predictor can effectively distinguish stemness subtypes

Consistently, 100 MLs and unsupervised hierarchical clustering based on the 9-gene stemness subtype predictor showed that our predictor is highly effective in accurately distinguishing the stemness subtypes and can be used as an alternative clinically practical marker panel instead of the 18 stemness signatures (Additional file 1: Fig. S11a–d, Remark E).

#### Stemness subtype predictor has excellent performance in predicting malignancy, recurrence, progression, metastasis, and treatment response

Subsequently, we investigated the performance of our predictor in distinguishing malignancy and predicting tumor recurrence, progression, metastasis, and ADT efficacy. In TCGA-PRAD, GSE21034 [32], and GSE70770 [38] datasets, the average area under the receiver operating characteristic curves (AUCs) of the top 10 MLs for identifying benign and malignant tumors were 89.8%, 89.5%, and 89.4%, respectively (Additional file 1: Fig. S11e). For the prediction of ADT response, the top 10 MLs had mean AUCs of 88.6%, 80.5%, and 76.5% in GSE6811 [62], GSE28680 [63], and Alumkal_2020 [64], respectively (Fig. 5e, Additional file 1: Fig. S11f). For the prediction of biochemical recurrence, the mean AUCs of the top 10 MLs in prostate_dkfz_2018 [37], GSE70770 [38], and GSE21034 [32] were 78.9%, 74%, and 72.8%, respectively (Fig. 5e, Additional file 1: Fig. S11f). In predicting metastasis, the mean AUCs were 89.4% in GSE21034 [32], 74.3% in GSE116918 [65] (Fig. 5e), and 66.3% in GSE46691 [66] (Additional file 1: Fig. S11f). The average AUC for the prediction of progression was 65.8% (Fig. 5e). Detailed data of the above results can be found in Additional file 2: Data S2.

#### Stemness subtype predictor can serve as a prognostic risk model

We calculated stemness-related risk scores (SRS) for each patient based on the above nine genes. Samples were then categorized into high and low SRS groups, and K–M analysis showed that the high SRS group had significantly worse PFI, OS and DFI than the low SRS group (Fig. 5f, Additional file 1: Fig. S11g–i). Univariate COX analysis revealed that SRS, Gleason score, pT, pN, and purity were the risk factors for PFI (Additional file 1: Figure S11j). Multivariate COX analysis further confirmed that SRS was the most significant independent prognostic indicator of PCA (Fig. 5g, HR = 2.04, p = 0.005). Furthermore, receiver operating characteristic (ROC) analysis demonstrated that SRS can serve as an effective clinical prognostic biomarker (Additional file 1: Fig. S11k–o). The K–M analysis based on another six independent PCA datasets [32, 37, 39, 67–69] further validated the above results (Additional file 1: Fig. S11p–u, Remark E).

### Conserved stemness subtypes in pan-tumors

#### Three stemness subtypes with significantly different prognoses are prevalent in pan-tumors

To determine whether our stemness subtypes were conserved in pan-tumors, we downloaded four pan-tumor datasets: TcgaTargetGtex, PCAWG, ICGC, and GSE2109. Using the 18 stemness signatures, we consistently classified the datasets into three stemness subtypes, each with significantly different prognoses (Additional file 1: Fig. S12a–h). This suggests that stemness subtypes have a general commonality in pan-tumors.

Furthermore, we confirmed that the stemness subtype predictor can effectively distinguish pan-tumor stemness subtypes, and SRS can predict the clinical prognosis of pan-tumor patients (Additional file 1: Fig. S12i–s, Remark F; Additional file 2: Data S2).

#### HS patients are more inclined to benefit from immunotherapy

**Fig. 6:**
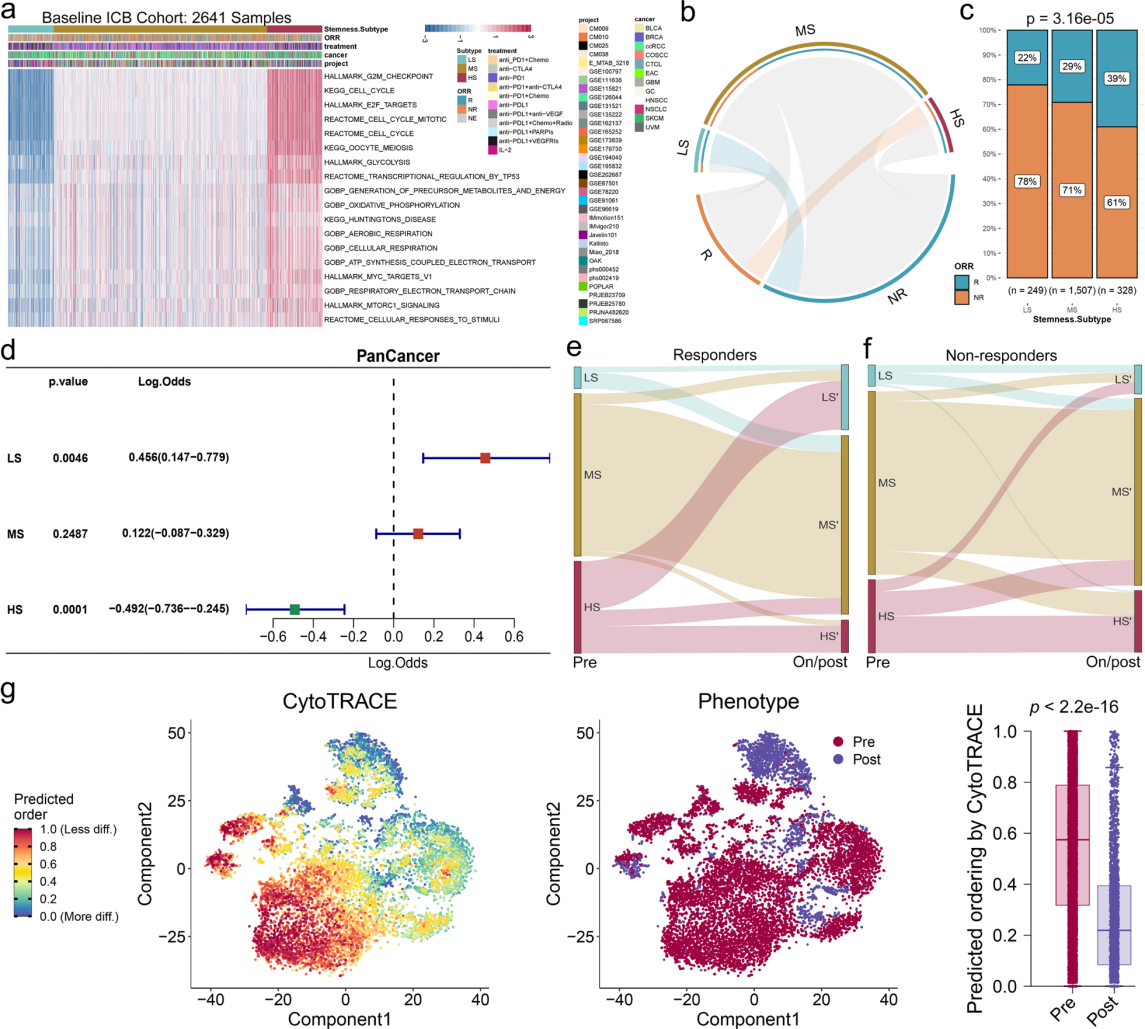
Interactions between stemness subtypes and ICB treatment in pan-tumors.** a** Unsupervised hierarchical clustering based on the stemness activity scores of the 18 stemness signatures clustered the baseline pan-tumor patients treated with ICB into three subtypes. **b** Hypergeometric test collaborates an association between stemness subtypes of ICB pan-tumors and responsiveness of ICB therapy. **c** Stacked histogram showing differences in responsiveness of the three pan-tumor stemness subtypes to ICB. **d** Univariate logistic regression shows the effect of the three stemness subtypes on ICB efficacy. **e**, **f** Sankey diagram showing sample flow for pre-treatment and on/post-treatment of ICB. Separate presentation for responders (**e**) and non-responders (**f**). **g** t-SNE plot of pre-treatment and post-treatment tumor cells of head and neck squamous cell carcinoma (HNSCC, medium), along with their corresponding cytoTRACE scores (left), and the comparison of these scores between two groups (right)

Here, we investigated the correlation between stemness subtypes and ICB responsiveness in pan-tumors. We collected RNA-seq data from 2641 pretreatment samples from patients across 36 datasets and 12 tumor types who received ICB therapy [70–102]. Figure 6a clearly showed that these samples could be classified into three subtypes based on 18 stemness signatures. Hypergeometric test showed that stemness subtypes were significantly correlated with ICB responsiveness (Fig. 6b). The response rate increased progressively across the three subtypes (Fig. 6c). These results were further corroborated by K–M analysis, submap analysis [55], and separate clustering analysis of each cancer type (sample size > 50) (Additional file 1: Fig. S13a–g, Remark G).

Subsequently, univariate logistic regression analysis was performed based on the RNA-seq data of pre-ICB treatment samples to evaluate the effect of the three stemness subtypes on immunotherapy outcomes. Our findings revealed LS as a risk factor and HS as a promoting factor for response (Fig. 6d). Similar observations were also made in separate analyses of bladder cancer (BLCA), breast cancer (BRCA), non-small cell lung cancer (NSCLC), melanoma (SKCM), and head and neck squamous cell carcinoma (HNSCC) (Additional file 1: Fig. S13h). Nonetheless, the stemness subtypes did not significantly affect immunotherapy in clear cell renal cell carcinoma (ccRCC) (Additional file 1: Fig. S13h). We further compared the stemness subtypes with other ICB predictive biomarkers using the IMvigor210 dataset. The results showed that the stemness subtype was an effective predictor of ICB responsiveness (Additional file 1: Fig. S13i–l, Remark G).

Next, we attempted to further explore the effect of immunotherapy on stemness. We collected bulk RNA-seq data of paired samples from the same patients before and on/post ICB treatment [80, 82, 86, 93, 96, 98–100, 102]. Using hierarchical clustering to distinguish these samples into three subtypes (Additional file 1: Fig. S14a), we found that the proportion of HS decreased significantly after ICB therapy, while the proportion of LS increased significantly (Additional file 1: Fig. S14b). Further distinguishing between responders and non-responders, we found that most of the HS patients who responded to ICB treatment shifted to LS after ICB treatment (Fig. 6e, Additional file 1: Fig. S14c), while most of the HS patients who were resistant remained unchanged after treatment (Fig. 6f, Additional file 1: Fig. S14d). These observations were consistent across analyses of HNSCC, esophageal adenocarcinoma (EAC), and SKCM (Additional file 1: Fig. S14e–g). We also validated our findings using a single-cell dataset of HNSCC, which demonstrated a significant decrease in cytoTRACE scores following ICB treatment (Fig. 6g).

## Discussion

We applied single-cell and bulk RNA/DNA-seq technologies to evaluate the stemness status of patients based on stemness signatures. This allowed us to subtype the patients into three stemness subtypes. Specifically, HS patients are sensitive to androgen deprivation therapy, taxanes, and immunotherapy and have the highest stemness, malignancy, TMB levels, worst prognosis, and immunosuppression. LS patients are sensitive to platinum-based chemotherapy but resistant to immunotherapy and have the lowest stemness, malignancy, and TMB levels, best prognosis, and the highest immune infiltration. MS patients represent an intermediate status of stemness, malignancy, and TMB levels with a moderate prognosis. Our 9-gene stemness subtype predictor demonstrated high sensitivity and specificity, and could be easily used to identify stemness subtypes of patients through real-time quantitative PCR or IHC. This simple procedure provides a powerful tool for clinical tumor typing and treatment selection.

PCSCs have been shown to play a significant role in the occurrence, development, treatment resistance, and recurrence of PCA [21]. In our study, the HS subgroup exhibited a significantly worse prognosis (Fig. 2e), significantly higher Gleason score, and expressed the highest level of serum PSA (Fig. 3c), indicating the highest malignancy in HS patients. It is essential to study the signaling pathways and functional annotations associated with PCSCs to understand the molecular mechanisms of carcinogenesis and to identify potential drug targets. Several signaling pathways are closely related to the regulation of PCSCs, such as the PI3K/AKT/mTOR pathway and the Wnt/β-catenin pathway [103, 104]. Studies have shown that PCSCs have a relatively slow proliferation rate compared to ordinary PCA cells and exist in a quiescent status [105, 106]. GSVA and GSEA revealed that cell cycle-related signaling pathways, including cell cycle checkpoints, were enriched in HS (Additional file 1: Fig. S8a, b). However, IPA showed that the cell growth, proliferation, and development of HS were significantly inhibited (Additional file 1: Fig. S8e). These results suggested that while the signaling pathways and molecules related to the cell cycle are upregulated in HS, their proliferative activity is suppressed or static. This phenomenon may be attributed to the self-renewal and multidirectional differentiation ability of PCSCs, such as negative feedback regulation after excessive proliferation (enrichment of cell cycle checkpoints) and activation disorders of cell cycle-related proteins [106–109].

The PCA treatment continues to be a major research focus. Radical prostatectomy and radiation therapy, with or without ADT, are the standard treatments for localized PCA. ADT remains the primary treatment for advanced disease [10, 110]. Although many treatments initially eradicate cancer cells, cancer often recurs owing to the presence of resistant PCSCs [111]. Therefore, targeting CSCs is critical for preventing tumor recurrence. The significance of molecular subtyping in tumors lies in its ability to help doctors better understand the biological characteristics, molecular mechanisms, development trends, and treatment responses of tumors, and to provide personalized treatment plans for patients. Existing molecular subtyping methods for PCA include the PAM50 method [18], which is used to guide ADT, and the Decipher method [19], which is used to guide radiotherapy and surgical treatment. By contrast, our stemness subtyping method can be used to guide various treatments. We found that different stemness subtypes exhibited different sensitivities to drugs. HS was more sensitive to ADT (bicalutamide), PARP inhibitors (olaparib), EGFR inhibitors (sunitinib and sorafenib), immunotherapy, and chemotherapeutic drugs such as docetaxel, etoposide, and gemcitabine. In contrast, LS was more sensitive to platinum drugs, erlotinib, and CDK inhibitors (AZD5438) (Fig. 4, Additional file 1: Fig. S9). Previously, the effectiveness of immunotherapy for PCA has been limited. However, with the development of immunology and cutting-edge molecular diagnostic tools, immunotherapy is expected to become a viable treatment option for PCA [112]. Unlike tumors, such as melanoma, bladder cancer, and NSCLC, which are highly responsive to immunotherapy and characterized by infiltrating lymphocyte proliferation, PCA is considered a “cold” tumor with an immunosuppressive TME [9, 112]. Although the effectiveness of ICB monotherapy for PCA is limited, combined strategies with other standard treatments (ADT, chemotherapy, PARP inhibitors, radium-223, and tyrosine kinase inhibitors) have shown some positive effects [9]. Overall, these findings underscore the importance of molecular subtyping for guiding cancer treatment. By tailoring therapies to the specific molecular characteristics of tumors, doctors can improve treatment outcomes and help patients achieve the best possible outcomes.

The significance of pan-cancer research lies in the application of diagnosis and treatment to more tumors through cross-tumor similarities [113]. We validated the conserved characteristics of the three subtypes in pan-tumors and their responsiveness to immunotherapy using 28,381 pan-tumor samples and 2641 ICB pretreatment samples. This indicates that our findings have implications beyond PCA, and can potentially benefit a wider range of patients. Furthermore, developing a predictive model using multiple MLs and datasets can enhance the model’s generalization ability and prevent overfitting or underfitting issues, which is an effective way to improve the accuracy and robustness of the predictors. We jointly developed a 9-gene stemness subtype predictor with high sensitivity, specificity, and excellent generalization ability using four datasets and 76 machine learning algorithms. Significantly, this predictor can be further developed into a kit for clinical application. We believe that this predictor has great potential for clinical application, as it offers rapid and reliable molecular diagnosis and prognosis for patients with PCA and guides personalized treatment decisions. Moreover, this predictor can facilitate the enrollment of PCA patients into clinical trials for immunotherapy or other targeted therapies based on their stemness subtype, and it may also be applicable to other cancer types with the same stemness subtypes as PCA.

Although this study yielded valuable insights, it is important to acknowledge some of its limitations. First, the sample size for our real-world validation was only 60, and we did not include any pan-cancer samples. Expanding the sample size and including pan-cancer samples for verification in future studies are essential. Second, there is a lack of RNA-seq data of PCA treated with ICB. Although the pan-tumor ICB treatment cohort validated the relationship between stemness subtypes and immunotherapy responsiveness, further validation is still needed for PCA immunotherapy datasets.

## Conclusions

In conclusion, tumor molecular typing is of great significance for understanding how cancer develops and progresses, as well as for guiding clinical treatment and the development of new anticancer drugs. By categorizing PCA patients into three stemness subtypes, we can systematically characterize patients from various points of view, e.g., stemness, prognosis, clinical pathological features, mutation patterns, malignancy degree, immune infiltration levels, and efficiencies of different treatments, including immunotherapy. This classification method is also applicable to pan-tumor analyses. Furthermore, the 9-gene stemness subtype predictor we developed is expected to be a clinically useful tool for precision oncology. Significantly, our method provides a pipeline for the development of cancer classification that can be applied to various tumors based on different research hotspots.

## Methods

### Patients and datasets collection

Five PCA scRNA-seq datasets [25–29], 20 PCA bulk RNA-seq datasets [32–40, 45, 62–69], four bulk-RNA-seq datasets of pan-tumors, one ICB-treated bulk RNA-seq cohort of pan-tumors (consisting of 36 independent datasets) [70–102], a pluripotent stem cell (PSC) expression matrix, and DNA methylation, somatic mutation, and copy number alteration data from TCGA-PRAD were used in this study. The database used in our study included the MSigDB [114] (v2023.1.Hs, http://www.gsea-msigdb.org/gsea/index.jsp), and STRING [115] (v11.5, https://cn.string-db.org/). See Additional file 1: Methods S1 and Table S1 for more details.

### Human samples

After obtaining patient consent and approval from the institutional research ethics committee, we collected paraffin-embedded tissue sections of prostate cancer and benign prostatic hyperplasia (BPH) from the Pathology Department of the Shanghai Sixth People’s Hospital.

### Stemness analysis

The cytoTRACE (v0.3.3) package [30] was used to calculate cytoTRACE scores for stemness evaluation in single-cell RNA-seq data. The OCLR machine learning algorithm [31] quantified stemness levels using mRNAsi, mDNAsi, EREG_mDNAsi, and DMPsi scores in bulk RNA-seq data. Higher scores indicate greater stemness levels. In K–M analysis, the surv_cutpoint function in the Survminer (v0.4.9) package calculated the optimal cutoff point to divide patients into high and low stemness groups. See Additional file 1: Methods S1 for further details.

### Clustering analysis

Unsupervised hierarchical clustering was used for clustering analysis based on stemness signatures, implemented using the hclust function. Consensus clustering and NMF were employed using the ConsensusClusterPlus [44] (v1.58.0) and NMF [43] (v0.24.0) packages. The optimal number of clusters was determined using the consensus heatmap, cumulative distribution function (CDF) curves, and the proportion of ambiguous clustering algorithm (PAC) [116] (Additional file 1: Fig. S6a). See Additional file 1: Methods S1 for further details.

### Survival analysis

Survival analysis was conducted using the Survival (v3.4.0) and Survminer (v0.4.9) packages. The Kaplan–Meier method plotted the survival curve, and the log-rank test compared survival differences between groups. The Cox proportional hazards model investigated covariate effects on survival time, calculating the risk ratio and confidence interval.

Additionally, the timeROC package (v0.4) was used to estimate the time-dependent ROC curve and AUC, which allowed us to evaluate the prognostic ability of SRS.

### Identification of stemness marker genes and developing of signatures for stemness classifications

The identification process is illustrated in Additional file 1: Fig. S5a. We utilized scRNA-seq data to identify stemness marker genes in malignant and high-grade PCA epithelial cells, and used bulk RNA-seq and DNA methylation data to identify stemness marker genes in malignant PCA samples. Our approach involved analyzing multiple datasets, performing Spearman correlation analysis and DE analysis, and selecting genes that were positively correlated with stemness scores and were upregulated in malignant and high-grade PCA. We then analyzed gene sets on MSigDB [114] with the stemness marker genes obtained and identified significant risk signaling pathways using univariate COX analysis for subsequent stemness classification. See Additional file 1: Methods S1 for further details.

### Immunohistochemistry (IHC)

Paraffin sections underwent dewaxing, antigen retrieval, and serum blocking. They were incubated with primary antibodies overnight at 4 °C, secondary antibodies and SABC for 30 min at 37 °C. Sections were stained with DAB and counterstained with hematoxylin. The primary antibodies used were the NCAPG Polyclonal antibody (24563-1-AP, Proteintech, Wuhan, China), HMMR-specific polyclonal antibody (15820-1-AP, Proteintech, Wuhan, China), HURP polyclonal antibody (12038-1-AP, Abcam, Proteintech, Wuhan, China), and SKA3 antibody (SC-390326, Santa, California, USA).

### Statistical analysis

Numerical variables were compared using t-tests or ANOVA, categorical variables using χ^2^, Fisher’s exact or Kruskal–Wallis tests. Non-normally distributed variables were compared using non-parametric tests. Correlations were evaluated using Pearson or Spearman tests. Survival differences were compared using the log-rank test. Confidence intervals (CIs) were reported as 95% and significance was set at P < 0.05. Analyses were performed using R (v4.2.1), Python (v3.10), and Origin 2022 software.

More methods and details can be seen in Additional file 1: Methods S1.

### Supplementary Information

**Additional file 1: Table S1. **Characteristics of scRNA-seq datasets and bulk RNA-seq datasets enrolled in this study. **Table S2.** Details of 9-gene stemness subtype predictor. **Figure S1.** The holistic design of the current study. **Figure S2.** Uniform manifold approximation and projection (UMAP) of all cells captured across 5 single-cell RNA-seq (scRNA-seq) datasets. **Figure S3.** Correlation of stemness levels with clinical, pathological, and molecular features in patients with prostate cancer (PCA). **Figure S4.** Correlation of stemness levels with tumor immune microenvironment (TIME) in PCA patients. **Figure S5.** Development of 18 signatures for stemness classification. **Figure S6.** Identification of three PCA stemness subtypes based on stemness signatures. **Figure S7.** Comparison of molecular features among three PCA stemness subtypes. **Figure S8.** Signaling pathways and functional annotations of three PCA stemness subtypes. **Figure S9.** Comparison of drug sensitivities and TIME patterns among three PCA stem subtypes. **Figure S10.** Construction of stemness subtype predictor. **Figure S11.** Validation of stemness subtype predictor. **Figure S12.** Conservation of the three stemness subtypes in pan-tumors. **Figure S13.** The impact of three pan-cancer stemness subtypes on ICB treatment and their predictive performance for ICB efficacy. Additional Remarks A-G. Methods S1.

**Additional file 2: Data S1.** Eighteen stemness signatures for stemness classification. **Data S2.** Prediction performances of 9- gene stemness subtype predictor based on 100 machine learning algorithms (MLs). **Data S3.** Details of 100 machine learning algorithms.

## Data Availability

The original contributions presented in the study are included in the article/Additional files. Further inquiries can be directed to the corresponding authors. The computer R codes for the processing and analysis of this study are available at https://github.com/Hao-Zou-lab/Stemness.Subtype.

## Abbreviations

PCA: Prostate cancer
LS: Low stemness
MS: Medium stemness
HS: High stemness
TMB: Tumor mutation load
ICB: Immune checkpoint blockade
PSA: Prostate-specific antigen
CSCs: Cancer stem cells
scRNA-seq: Single-cell RNA sequencing
IHC: Immunohistochemistry
MLs: Machine learning algorithms
ADT: Androgen deprivation therapy
GS: Gleason score
OCLR: One-class logistic regression
TCGA-PRAD: The Cancer Genome Atlas-Prostate Adenocarcinoma
AR: Androgen receptor
pT: Pathological T stage
SCNA: Somatic copy number alteration
FGA: Fragment genomic alteration
K–M analysis: Kaplan–Meier analysis
OS: Overall survival
PFS: Progression-free survival
DFS: Disease-free survival
DFI: Disease-specific survival
TIME: Tumor immune microenvironment
High.immu: High immune infiltration;
Low.immu: Low immune infiltration
WCDT: West Coast Dream Team
CNAs: Copy number alterations
Hetloss: Heterozygously deleted
Homdel: Homozygously deleted
GSVA: Gene set variation analysis
GSEA: Gene set enrichment analysis
IPA: Ingenuity pathway analysis
AUC: Area under the receiver operating characteristic curve
WGCNA: Weighted gene co-expression network analysis
PPI: Protein–protein interaction
SRS: Stemness-related risk scores
PR: Radical prostatectomy
PSCs: Pluripotent stem cells
GEO: Gene Expression Omnibus
PCBC: Progenitor Cell Biology Consortium
dbGaP: Database of Genotypes and Phenotypes
NCBI: National Center for Biotechnology Information
EGA: European Genome-Phenome Archive
BPH: Benign prostatic hyperplasia
CDF: Cumulative distribution function
PAC: Proportion of ambiguous clustering algorithm
TOM: Topological overlap matrix
TME: Tumor microenvironment
