# Supplementary material for: Integrative multi-omics analysis unveils stemness-associated molecular subtypes in prostate cancer and pan-cancer: prognostic and therapeutic significance
Source: J Transl Med. 2023 Nov 7;21:789. doi: 10.1186/s12967-023-04683-6 (PMC10629187; doi:10.1186/s12967-023-04683-6)
Supplement: Supplementary file 1 — Additional file 1: Table S1. Characteristics of scRNA-seq datasets and bulk RNA-seq datasets enrolled in this study. Table S2. Details of 9-gene stemness subtype predictor. Figure S1. The holistic design of the current study. Figure S2. Uniform manifold approximation and projection (UMAP) of all cells captured across 5 single-cell RNA-seq (scRNA-seq) datasets. Figure S3. Correlation of stemness levels with clinical, pathological, and molecular features in patients with prostate cancer (PCA). Figure S4. Correlation of stemness levels with tumor immune microenvironment (TIME) in PCA patients. Figure S5. Development of 18 signatures for stemness classification. Figure S6. Identification of three PCA stemness subtypes based on stemness signatures. Figure S7. Comparison of molecular features among three PCA stemness subtypes. Figure S8. Signaling pathways and functional annotations of three PCA stemness subtypes. Figure S9. Comparison of drug sensitivities and TIME patterns among three PCA stem subtypes. Figure S10. Construction of stemness subtype predictor. Figure S11. Validation of stemness subtype predictor. Figure S12. Conservation of the three stemness subtypes in pan-tumors. Figure S13. The impact of three pan-cancer stemness subtypes on ICB treatment and their predictive performance for ICB efficacy. Additional Remarks A-G. Methods S1. [file 12967_2023_4683_MOESM1_ESM.docx]

# Integrative Multi-Omics Analysis Unveils Stemness-Associated Molecular Subtypes in Prostate Cancer and Pan-Cancer: Prognostic and Therapeutic Significance

**Kun Zheng^1^, Youlong Hai^1^, Yue Xi^2^, Yukun Zhang^3^, Zheqi Liu^4^, Wantao Chen^5^, Xiaoyong Hu^1*^, Xin Zou^6, 7*^, Jie Hao^8*^**

*^1^Department of Urology, Shanghai Sixth People's Hospital Affiliated to Shanghai Jiao Tong University School of Medicine, 200233, Shanghai, China.*

*^2^Department of Reproductive Medicine, Central Hospital Affiliated to Shandong First Medical University, 250013, Jinan, Shandong, China*

*^3^Beijing University of Chinese Medicine East Hospital, Zaozhuang Hospital, 277000, Zaozhuang, Shandong, China.*

*^4^Department of Oral and Maxillofacial Surgery, Zhongshan Hospital, Fudan University, 200032, Shanghai, China.*

*^5^Ninth People’s Hospital, Shanghai Key Laboratory of Stomatology & Shanghai Research Institute of Stomatology, National Clinical Research Center of Stomatology, Shanghai Jiao Tong University School of Medicine, 200011, Shanghai, China.*

*^6^Jinshan Hospital Center for Tumor Diagnosis & Therapy, Jinshan Hospital, Fudan University, 201508, Shanghai, China.*

*^7^Department of Pathology, Jinshan Hospital, Fudan University, 201508, Shanghai, China.*

*^8^Institute of Clinical Science, Zhongshan Hospital, Fudan University, 200032, Shanghai, China.*

***Corresponding author**

Jie Hao, E-mail: [jhao@fudan.edu.cn](mailto:jhao@fudan.edu.cn);

Xin Zou, E-mail: xzou@fudan.edu.cn;

Xiaoyong Hu, E-mail: [huxiaoyong@sjtu.edu.cn](mailto:huxiaoyong@sjtu.edu.cn).

# Additional file 1

## Additional Tables

- Table S1
- Table S2

## Additional Figures

- Figure S1
- Figure S2
- Figure S3
- Figure S4
- Figure S5
- Figure S6
- Figure S7
- Figure S8
- Figure S9
- Figure S10
- Figure S11
- Figure S12
- Figure S13
- Figure S14

## Additional Remarks

- Remark A
- Remark B
- Remark C
- Remark D
- Remark E
- Remark F
- Remark G

## Additional Methods

- Patients and datasets collection
- Study Design
- Single-cell RNA-seq data processing and identification of epithelial cells
- Bulk RNA-seq data processing
- Differential expression (DE) analysis
- Stemness analysis
- Pathway analysis
- Clustering Analysis
- Weighted gene co-expression network analysis (WGCNA)
- Protein–protein interaction network analysis (PPI)
- Somatic mutation and copy number variation analysis
- Evaluation of immune patterns
- Evaluation of immunotherapy efficacy
- Evaluation of drug sensitivity
- Identification of stemness marker genes and developing of signatures for stemness classifications
- Construction and verification of stemness subtype predictor
- Calculation of SRS

## Additional References

Table S1. Characteristics of scRNA-seq datasets and bulk RNA-seq datasets enrolled in this study.

**a. Single-cell RNA-seq datasets of prostate cancer**

| **Datasets** | **Patients** | **Samples** | | **Epithelial cells** | **Age (yr)** | **PSA (ng/ml)** | **pT** | | **pN** | | | **Grade** | | **Ref.** | **Download** |
| --- | --- | --- | --- | --- | --- | --- | --- | --- | --- | --- | --- | --- | --- | --- | --- |
|  |  | **T** | **N** |  |  |  | **pT2** | **pT3** | **pN0** | **pN1** | **Nx** | **Low** | **High** |  |  |
| **GSE193337** | 4 | 4 | 4 | 9014 | 67 (61-73) | 9.7 (2.3-20) | 2 | 2 | 4 |  |  |  |  | [1] | [GEO](https://www.ncbi.nlm.nih.gov/geo/) |
| **GSE185344** | 7 | 7 | 7 | 17687 | 65 (52-74) | 10 (3.1-18.1) |  | 7 | 5 |  | 2 | 6 | 1 | [2] | [GEO](https://www.ncbi.nlm.nih.gov/geo/) |
| **GSE176031** | 11 | 27 | 8 | 14339 |  | 8.5 (3.9-19.6) |  |  |  |  |  | 10 | 1 | [3] | [GEO](https://www.ncbi.nlm.nih.gov/geo/) |
| **prostate_portal_300921** | 10 | 10 | 10 | 10587 |  | 23.2 (3.5-149) |  |  |  |  |  |  |  | [4] | [Prostate Cell Atlas](https://www.prostatecellatlas.org/) |
| **GSE141445** | 13 | 13 |  | 23723 | 73 (61-81) | 53.2 (7.1-336) | 5 | 8 | 9 | 4 |  | 4 | 9 | [5] | [GEO](https://www.ncbi.nlm.nih.gov/geo/) |

**b. Bulk RNA-seq datasets of prostate cancer**

| **Datasets** | **Samples** | | **Age (yr)** | **PSA (ng/ml)** | **pT/cT** | | | | **pN/cN** | | **pM/cM** | | **Grade** | | **Survival** | **TMB** | **Purity** | **Ref.** | **Download** |
| --- | --- | --- | --- | --- | --- | --- | --- | --- | --- | --- | --- | --- | --- | --- | --- | --- | --- | --- | --- |
|  | **T** | **N** |  |  | **T1** | **T2** | **T3** | **T4** | **N0** | **N1** | **M0** | **M1** | **Low** | **High** |  |  |  |  |  |
| **TCGA-PRAD** | 501 | 52 | 61 (41-78) | 10.9 (1.6-87) |  | 189 | 294 | 11 | 348 | 80 | 457 | 3 | 248 | 88 | OS, PFI, DFI, DSS | √ | √ |  | [TCGA](https://portal.gdc.cancer.gov/) |
| **GSE35988** | 76 | 12 |  |  |  |  |  |  |  |  |  |  |  |  |  |  |  | [6] | [GEO](https://www.ncbi.nlm.nih.gov/geo/) |
| **GSE94767** | 185 | 51 |  | 9.4 (3.1-40) | 1 | 91 | 89 | 4 |  |  |  |  | 168 | 17 | BCR |  | √ | [7] | [GEO](https://www.ncbi.nlm.nih.gov/geo/) |
| **GSE134051** | 216 | 39 |  |  |  |  |  |  |  |  |  |  | 103 | 113 |  |  |  | [8] | [GEO](https://www.ncbi.nlm.nih.gov/geo/) |
| **GSE183019** | 84 | 84 | 63 (51-75) | 7.2 (1.6-43.6) |  | 43 | 41 |  |  |  |  |  | 80 | 4 |  |  |  |  | [GEO](https://www.ncbi.nlm.nih.gov/geo/) |
| **GSE21034** | 150 | 29 | 58 (37-83) | 16.1 (1.1-506) |  | 86 | 47 | 8 | 105 | 16 | 122 | 28 | 130 | 19 | OS, BCR |  |  | [9] | [GEO](https://www.ncbi.nlm.nih.gov/geo/) |
| **GSE6099** | 52 | 52 |  |  |  |  |  |  |  |  |  |  |  |  |  |  |  | [10] | [GEO](https://www.ncbi.nlm.nih.gov/geo/) |
| **prad_su2c_2019** | 328 |  | 61 (39-86) | 108.5 (0.2-2800) |  |  |  |  |  |  |  | 328 | 103 | 159 | OS | √ |  | [11] | [cBioPortal](https://www.cbioportal.org/) |
| **WCDT** | 99 |  | 71 (45-90) | 158.4 (0.4-1500) |  |  |  |  |  | 37 |  | 99 | 24 | 36 | OS |  |  | [12] | [WCDT](http://davidquigley.com/prostate.html) |
| **prostate_dkfz_2018** | 118 |  | 47 (32-52) | 30.8 (1.9-743) |  | 74 | 35 | 7 | 118 |  | 118 |  | 100 | 18 | BCR | √ | √ | [13] | [cBioPortal](https://www.cbioportal.org/) |
| **GSE70770** | 220 | 73 | 62 (41-93) | 15.2 (1.5-282) |  | 83 | 118 | 1 | 99 | 8 | 35 | 7 | 180 | 33 | OS, BCR |  | √ | [14] | [GEO](https://www.ncbi.nlm.nih.gov/geo/) |
| **GSE46602** | 36 | 14 | 62 (46-71) | 18.2 (5.3-42.5) |  | 19 | 17 |  |  |  |  |  | 32 | 4 | BCR |  |  | [15] | [GEO](https://www.ncbi.nlm.nih.gov/geo/) |
| **GSE54460** | 106 |  |  | 10.9 (1.8-72.6) | 14 | 73 | 17 | 1 |  |  |  |  | 91 | 15 | BCR |  | √ | [16] | [GEO](https://www.ncbi.nlm.nih.gov/geo/) |
| **GSE84042** | 73 |  | 60 (44-72) | 7.8 (1.7-39.5) |  | 40 | 33 |  |  |  |  |  |  |  | BCR |  | √ | [17] | [GEO](https://www.ncbi.nlm.nih.gov/geo/) |
| **GSE116918** | 248 |  | 67 (48-79) | 25.5 (3.2-222.3) | 51 | 76 | 92 | 4 |  |  | 226 | 22 | 141 | 107 | BCR, MFS |  |  | [18] | [GEO](https://www.ncbi.nlm.nih.gov/geo/) |
| **E-MTAB-6128** | 101 | 40 | 63 (46-76) | 8.2 (1.6-57) |  | 69 | 30 | 1 |  |  |  |  | 82 | 18 | BCR |  | √ | [19] | [ArrayExpress](https://www.ebi.ac.uk/arrayexpress/) |
| **Alumkal_2020** | 25 |  |  |  |  |  |  |  |  |  |  |  |  |  |  |  |  | [20] | Supplements |
| **GSE6811** | 35 |  |  |  |  |  |  |  |  |  |  |  |  |  |  |  |  | [21] | [GEO](https://www.ncbi.nlm.nih.gov/geo/) |
| **GSE28680** | 20 | 4 |  |  |  |  |  |  |  |  |  |  |  |  |  |  |  | [22] | [GEO](https://www.ncbi.nlm.nih.gov/geo/) |
| **GSE46691** | 545 |  |  |  |  |  |  |  |  |  | 333 | 212 | 334 | 211 |  |  |  | [23] | [GEO](https://www.ncbi.nlm.nih.gov/geo/) |

**c. Bulk RNA-seq datasets of pan-tumors**

| **Datasets** | **Samples** | | | **Age (yr)** | **Tissue** | **Cancer type** | **Gender** | | **Survival** | **Download** |
| --- | --- | --- | --- | --- | --- | --- | --- | --- | --- | --- |
|  | **Total** | **T** | **N** |  |  |  | **Male** | **Female** |  |  |
| **TcgaTargetGtex** | 16011 | 10530 | 5481 |  | 29 | 38 | 8581 | 7272 | OS, PFI, DFI, DSS | [UCSC Xena](https://xenabrowser.net/datapages/) |
| **PCAWG** | 1466 | 1305 | 161 | 61 (17-90) | 22 | 29 | 691 | 654 | OS | [UCSC Xena](https://xenabrowser.net/datapages/) |
| **ICGC** | 8746 | 8097 | 649 | 60 (14-90) | 16 | 21 | 4030 | 4716 | OS | [UCSC Xena](https://xenabrowser.net/datapages/) |
| **GSE2109** | 2158 | 2158 |  |  | 192 |  | 697 | 1458 |  | [GEO](https://www.ncbi.nlm.nih.gov/geo/) |

**d. Baseline bulk RNA-seq datasets of pan-tumors treated with immune-checkpoint blockade therapy**

| **Datasets** | **Patients** | **Cancer type** | **Treatment** | **Response** | | | **OS** | **PFS** | **Gender** | | **Ref.** | **Download** |
| --- | --- | --- | --- | --- | --- | --- | --- | --- | --- | --- | --- | --- |
|  |  |  |  | **R** | **NR** | **NE** |  |  | **Male** | **Female** |  |  |
| **IMvigor210** | 348 | BLCA | anti-PDL1 | 68 | 230 | 50 | √ |  | 272 | 76 | [24] | [R package](http://research-pub.gene.com/IMvigor210CoreBiologies/#transcriptome-wide-gene-expression-data) |
| **Kallisto** | 25 | BLCA | anti-PDL1 | 7 | 14 | 4 | √ | √ | 22 | 3 | [25] | [zenodo](https://zenodo.org/record/546110) |
| **GSE111636** | 11 | BLCA | anti-PD1 | 6 | 5 |  |  |  |  |  |  | [GEO](https://www.ncbi.nlm.nih.gov/geo/) |
| **GSE173839** | 71 | BRCA | anti-PDL1+PARPIs | 29 | 42 |  |  |  |  | 71 | [26] | [GEO](https://www.ncbi.nlm.nih.gov/geo/) |
| **GSE194040** | 69 | BRCA | anti-PD1+Chemotherapy | 31 | 38 |  |  |  |  | 69 | [27] | [GEO](https://www.ncbi.nlm.nih.gov/geo/) |
| **phs002419** | 14 | BRCA | anti-PD1+Chemotherapy | 4 | 9 | 1 | √ | √ |  | 14 | [28] | [dbGaP](https://www.ncbi.nlm.nih.gov/gap/) |
| **Checkmate009** | 16 | KIRC | anti-PD1 | 3 | 13 |  | √ | √ | 13 | 3 | [29, 30] | [ArrayExpress](https://www.ebi.ac.uk/arrayexpress/) |
| **Checkmate010** | 45 | KIRC | anti-PD1 | 11 | 34 |  | √ | √ | 30 | 15 | [30, 31] | Supplements |
| **Checkmate025** | 120 | KIRC | anti-PD1 | 25 | 86 | 9 | √ | √ | 94 | 26 | [30, 32] | [EGA](https://ega-archive.org/) |
| **E_MTAB_3218** | 59 | KIRC | anti-PD1 | 13 | 43 | 3 | √ | √ | 39 | 20 | [33] | [ArrayExpress](https://www.ebi.ac.uk/arrayexpress/) |
| **Miao_2018** | 33 | KIRC | anti-PD1/anti-PDL1/anti-PD1+anti-CTLA4 | 8 | 25 |  | √ | √ | 24 | 9 | [34] |  |
| **GSE67501** | 11 | KIRC | anti-PD1 | 4 | 7 |  |  |  | 7 | 4 | [35] | [GEO](https://www.ncbi.nlm.nih.gov/geo/) |
| **IMmotion151** | 407 | KIRC | anti-PDL1+anti-VEGF | 150 | 230 | 27 |  | √ | 281 | 126 | [36] | [EGA](https://ega-archive.org/) |
| **Javelin101** | 354 | KIRC | anti-PDL1+VEGFRIs |  |  |  |  | √ | 257 | 97 | [37] | Supplements |
| **GSE179730** | 11 | COSCC | anti-PD1 | 3 | 8 |  | √ | √ |  |  | [38] | [GEO](https://www.ncbi.nlm.nih.gov/geo/) |
| **GSE162137** | 25 | CTCL | anti-PD1 | 11 | 14 |  |  |  |  |  | [39] | [GEO](https://www.ncbi.nlm.nih.gov/geo/) |
| **GSE165252** | 35 | EAC | anti-PD1+Chemoterapy+Radiotherapy | 12 | 20 | 3 |  |  |  |  | [40] | [GEO](https://www.ncbi.nlm.nih.gov/geo/) |
| **PRJNA482620** | 17 | GBM | anti-PD1 | 10 | 7 |  | √ |  |  |  | [41] | [NCBI](https://www.ncbi.nlm.nih.gov/gap/) |
| **PRJEB25780** | 45 | GC | anti-PD1 | 12 | 33 |  |  |  |  |  | [42] | [NCBI](https://www.ncbi.nlm.nih.gov/gap/) |
| **GSE195832** | 28 | HNSCC | anti-PD1 | 9 | 19 |  |  |  | 26 | 2 | [43] | [Mendeley](https://doi.org/10.17632/7yb7s9769c.1) Data |
| **TJ_Val** | 20 | HNSCC | anti-PD1 | 5 | 15 |  |  |  |  |  | [43] | [Mendeley](https://doi.org/10.17632/7yb7s9769c.1) Data |
| **GSE126044** | 16 | NSCLC | anti-PD1 | 5 | 11 |  |  |  |  |  | [44] | [GEO](https://www.ncbi.nlm.nih.gov/geo/) |
| **GSE135222** | 27 | NSCLC | anti-PD1 |  |  |  |  | √ | 22 | 5 | [45] | [GEO](https://www.ncbi.nlm.nih.gov/geo/) |
| **OAK** | 344 | NSCLC | anti-PDL1 | 48 | 270 | 26 | √ | √ | 219 | 125 | [46] | [EGA](https://ega-archive.org/) |
| **POPLAR** | 95 | NSCLC | anti-PDL1 | 13 | 74 | 8 | √ | √ | 68 | 27 | [46] | [EGA](https://ega-archive.org/) |
| **Checkmate038** | 49 | SKCM | anti-PD1 | 9 | 34 | 6 | √ | √ | 24 | 25 | [33] | [ArrayExpress](https://www.ebi.ac.uk/arrayexpress/) |
| **GSE115821** | 14 | SKCM | anti-PD1/ anti-PD1+anti-CTLA4/ anti-CTLA4 | 2 | 12 |  |  |  |  |  | [47] | [GEO](https://www.ncbi.nlm.nih.gov/geo/) |
| **GSE131521** | 17 | SKCM | anti-PD1 |  |  |  | √ |  |  |  | [48] | [GEO](https://www.ncbi.nlm.nih.gov/geo/) |
| **GSE78220** | 27 | SKCM | anti-PD1 | 15 | 12 |  | √ |  | 19 | 8 | [49] | [GEO](https://www.ncbi.nlm.nih.gov/geo/) |
| **GSE91061** | 51 | SKCM | anti-PD1 | 10 | 39 | 2 |  |  |  |  | [50] | [GEO](https://www.ncbi.nlm.nih.gov/geo/) |
| **phs000452** | 116 | SKCM | anti-PD1/ anti-CTLA4 | 48 | 68 |  | √ | √ | 67 | 49 | [51] | [dbGaP](https://www.ncbi.nlm.nih.gov/gap/) |
| **PRJEB23709** | 73 | SKCM | anti-PD1/ anti-PD1+anti-CTLA4 | 40 | 33 |  | √ | √ | 47 | 26 | [52] | [NCBI](https://www.ncbi.nlm.nih.gov/gap/) |
| **SRP067586** | 9 | SKCM | anti-CTLA4 | 4 | 5 |  | √ |  | 5 | 4 | [53] | [NCBI](https://www.ncbi.nlm.nih.gov/gap/) |
| **GSE100797** | 25 | SKCM | IL-2 | 10 | 15 |  | √ | √ |  |  | [54] | [GEO](https://www.ncbi.nlm.nih.gov/geo/) |
| **GSE96619** | 5 | SKCM | anti-PD1 | 2 | 3 |  |  |  |  |  | [55] | [GEO](https://www.ncbi.nlm.nih.gov/geo/) |
| **GSE202687** | 9 | UVM | anti-PD1+anti-CTLA4 |  | 9 |  |  |  |  |  | [56] | [GEO](https://www.ncbi.nlm.nih.gov/geo/) |

PSA = prostate specific antigen; TMB = tumor mutation load

pT/cT = pathological/clinical tumor stage

pN/cN = pathological/clinical lymph node status (X = not assessed, 0 = no lymph node metastases, 1 = lymph node metastases)

pM/cM = pathological/clinical metastasis status (X = not assessed, 0 = no distant metastases, 1 = distant metastases)

Grade: High (Gleason Score > 7), Low (Gleason Score ≤ 7)

Samples: T = tumor samples, N = benign samples

Survival: OS = overall survival, PFI = progression-free interval, DFI = disease-free interval, DSS = disease-specific survival,

BCR = biochemical recurrence-free survival, MFS = metastasis-free survival, PFS = progression-free interval

Cancer type: BLCA = bladder cancer, BRCA = breast cancer, KIRC = kidney clear cell carcinoma, COSCC = oral-cavity squamous cell carcinoma,

EAC = esophageal adenocarcinoma, GBM = Glioblastoma, GC = gastric cancer, HNSCC = head and neck squamous cell carcinoma,

NSCLC = non-small cell lung cancer, SKCM = melanoma, UVM = uveal melanoma, CTCL = cutaneous T cell lymphoma

Response: R = response, NR = non-response, NE = not evaluation

Table S2. Details of 9-gene stemness subtype predictor.

| **Symbol** | **Entrez Gene Name** | **Location** | **Type(s)** | **Biomarker Application** | **Drug(s)** |
| --- | --- | --- | --- | --- | --- |
| **BUB1** | BUB1 mitotic checkpoint serine/threonine kinase | Nucleus | kinase |  |  |
| **CDK1** | cyclin dependent kinase 1 | Nucleus | kinase | efficacy | AG 024322, aloisine A, AZD5438, dinaciclib, milciclib, staurosporine, zotiraciclib |
| **DLGAP5** | DLG associated protein 5 | Nucleus | other |  |  |
| **HMMR** | hyaluronan mediated motility receptor | Plasma Membrane | transmembrane receptor |  |  |
| **KIF4A** | kinesin family member 4A | Nucleus | other |  |  |
| **NCAPG** | non-SMC condensin I complex subunit G | Nucleus | other |  |  |
| **SKA3** | spindle and kinetochore associated complex subunit 3 | Nucleus | other |  |  |
| **TOP2A** | DNA topoisomerase II alpha | Nucleus | enzyme | diagnosis, efficacy, prognosis, response to therapy, unspecified application | Alemtuzumab, phosphate, bevacizumab, etoposide, bortezomib |
| **TPX2** | TPX2 microtubule nucleation factor | Nucleus | other |  |  |

Figure S1. The holistic design of the current study.

**Figure S2. Uniform manifold approximation and projection (UMAP) of all cells captured across 5 single-cell RNA-seq (scRNA-seq) datasets, colored by broad cell type.** NK, natural killer cell; SMC, smooth muscle cells; MNP, mononuclear phagocyte.

**Figure S3. Correlation of stemness levels with clinical, pathological, and molecular features in patients with prostate cancer (PCA).** Related to Figure 1. **(a-c)** t-distributed stochastic neighbor embedding (t-SNE) plot of malignant and benign epithelial cells from GSE185344 **(a)**, GSE176031 **(b)**, and prostate_portal_300921 **(c)** datasets **(medium)**, along with their corresponding stemness scores (cytoTRACE, **left**), and the comparison of these scores between two groups **(right)**. (**d**) t-SNE plot of high and low grade PCA cells from GSE176031 dataset **(medium)**, along with their corresponding cytoTRACE scores **(left)**, and the comparison of these scores between two groups **(right)**. **(e)** Comparison of stemness scores (EREG_mDNSsi, DMPsi) between benign and malignant prostate samples, as well as between high (Gleason score [GS] > 7) and low (GS < 7) grade PCA samples from TCGA-PRAD. **(f)** Comparison of stemness scores (mRNAsi) between benign and malignant prostate samples from GSE35988, GSE94767, GSE134051, GSE183019, GSE21034, and GSE6099. **(g-i)** Kaplan-Meier (K-M) analysis demonstrates a correlation between the stemness scores (mDNAsi **[g]**, EREG_mDNAsi **[h]**, DMPsi **[i]**) and the prognosis of PCA patients from TCGA-PRAD. OS, overall survival, PFI, progression-free interval, DFI, disease-free interval, DSS, disease-specific survival. Dashed line: median survival time. Color range: 95% confidence interval (CI). **(j)** K-M analysis demonstrates a correlation between the mRNAsi and the prognosis of PCA patients from GSE94767, GSE21034, prostate_portal_2018, GSE70770, prad_su2c_2019, and WCDT datasets. BCR, biochemical recurrence-free survival. **(k)** Correlation of mRNAsi with serum prostate antigen (PSA), androgen receptor (AR), tumor purity, pathological T-stage (pT), pathological N stage (pN) in PCA patients. **(l)** Correlation of mRNAsi with age, weight, fraction genome altered (FGA), tumor mutation burden (TMB), and somatic copy number alterations (SCNA) cluster in PCA patients.

**Figure S4. Correlation of stemness levels with tumor immune microenvironment (TIME) in PCA patients. (a)** Consensus clustering divided PCA patients from TCGA-PRAD into two subtypes according to the activity scores of 48 published immune signatures: low immune infiltration (Low.immu) and high immune infiltration (High.imu) subtypes. **(b)** Raincloudplot shows the tumor microenvironment (TME) scores (StromalScore, ImmuneScore, ESTIMATEScore) of PCA patients in two TIME subtypes.

**(c)** The association between TIME subtypes and mRNAsi groups, achieved by the hypergeometric test. The mRNAsi groups were dichotomized at the median mRNAsi scores **(Upper)**. Raincloud plot showing the stemness levels in PCA patients of the two TIME subtypes **(Under)**. **(d)** Correlation heatmaps showing correlations of stemness indices (mRNAsi, mDNAsi, EREG_mDNAsi, DMPsi) with clinical and molecular features and tumor microenvironment (TME) scores (Stromal, Immune, ESTIMATE scores), achieved by Pearson correlation analysis. Links showing the correlation between immunocyte abundance and stemness indices, TME scores, and clinical and molecular features, implemented by Mantel test. FGA, fraction genome altered. *p < 0.05, **p < 0.01, ***p < 0.001.

**Figure S5. Development of 18 signatures for stemness classification.** **(a)** The workflow of identification stemness marker genes. **(b)** Forest plot visualizes the results of univariate COX analysis of stemness signatures. **(c)** Correlation heatmap of 18 stemness signatures. These signatures are mainly divided into two categories: cell cycle-related and metabolic-related.

**Figure S6. Identification of three PCA stemness subtypes based on stemness signatures.** Related to Figure 3. **(a)** Connective matrix for k = 3, which was the optimal cluster number (**left**). CDF curves of the consensus score from k = 2 to 10 (**medium**). The relative change in the area under the CDF curve from k = 2 to 10 (**right**). **(b)** Levels and trends of stemness scores (mDNAsi, EREG_mDNAsi, DMPsi) and tumor purity within three stemness subtypes. **(c)** K-M analysis shows no significant differences in OS, DFI and DSS among the three stemness subtypes of TCGA-PRAD. **(d)** Unsupervised nonnegative matrix factorization (NMF) based on the activity scores of the 18 stemness signatures classified PCA patients from TCGA-PRAD into three subtypes: Low stemness (LS), Medium stemness (MS), and High stemness (HS) subtypes **(left)**. Levels and trends of mRNAsi within these stemness subtypes **(right)**. **(e)** K-M analysis of three stemness subtypes classified by NMF from TCGA-PRAD. **(f)** Consensus clustering based on the activity scores of the 18 stemness signatures classified PCA patients from TCGA-PRAD into three subtypes: LS, MS, and HS subtypes **(left)**. Levels and trends of mRNAsi within these stemness subtypes **(right)**. **(g)** K-M analysis of three stemness subtypes classified by CensusClusterPlus from TCGA-PRAD. **(h-n)** Unsupervised hierarchical clustering based on the activity scores of the 18 stemness signatures classified PCA patients from prostate_dkfz_2018 **(h)**, GSE70770 **(i)**, GSE21034 **(j)**, GSE54460 **(k)**, GSE94767 **(l)**, prad_su2c_2019 **(m)**, and WCDT **(n)** into three subtypes **(left)**. Levels and trends of mRNAsi within these stemness subtypes **(right)**. (**o-u**) K-M analysis of three stemness subtypes from prostate_dkfz_2018 **(o)**, GSE70770 **(p)**, GSE21034 **(q)**, GSE54460 **(r)**, GSE94767 **(s)**, prad_su2c_2019 **(t)**, WCDT **(u)** datasets. *p < 0.05, **p < 0.01, ***P < 0.001, ****p < 0.0001.

**Figure S7. Comparison of molecular features among three PCA stemness subtypes.** Related to Figure 4. **(a-c)** Heatmap showing mutually exclusive or co-occurring events among top mutated genes in LS **(a)**, MS **(b),** and HS **(c)** subtypes. **(d)** Stacked histograms showing comparisons of mutations and copy number alterations (CNAs) of PCA common biomarkers among the three stemness subtypes.

**Figure S8. Signaling pathways and functional annotations of three PCA stemness subtypes. (a)** Gene set variation analysis (GSVA) reveals the top 10 signaling pathways enriched in the three stemness subtypes. **(b-d)** Gene set enrichment analysis (GSEA) shows the top 10 signaling pathways enriched in HS **(b)**, LS **(c)**, and MS **(d)** subtype. **(e, g, i)** Ingenuity pathways analysis (IPA) demonstrates the activation or inhibition status of canonical signaling pathways for HS **(e)**, MS **(g)**, and LS **(i)** subtypes. **(f, h, j)** Graphical summary explains biomolecular interactions and biological processes in HS **(f)**, MS **(h)**, and LS **(j)** subtypes. **(k)** Heatmap showing associations between gene expression of the three stemness subtypes and cancer and biological function.

**Figure S9. Comparison of drug sensitivities and TIME patterns among three PCA stem subtypes.** Related to Figure 6. **(a-n)** Comparisons of sensitivities of three stemness subtypes to clinically preferred and recommended drugs. **(o)** Stacked histogram showing the compositional differences of the TIME subtypes between the three PCA stemness subtypes. **(p)** Boxplots showing comparisons of expression levels of immune checkpoint molecules among the three stemness subtypes. **(q)** Hypergeometric tests reveal an association between stemness subtypes and responsiveness of immune-checkpoint blockade (ICB) therapy. Evaluated by TIDE algorithm.

**Figure S10. Construction of stemness subtype predictor.** Related to Figure 7. **(a)** Development pipeline for the stemness subtype predictor. **(b-g)** Correlation analysis between module eigengenes and stemness subtypes of prostate_dkfz_2018 **(b)**, GSE70770 **(c)**, GSE21034 **(d)**, GSE54460 **(e)**, prad_su2c_2019 **(f)**, and WCDT **(g)** datasets. **(h-i)** Comparison of expression levels of 9 critical stemness subtype marker genes. Group by sample type **(h)** and GS **(i)**.

**Figure S11. Validation of stemness subtype predictor.** Related to Figure 7. **(a)** Radar graph displays the performance of the 9-gene stemness subtype predictor for predicting the HS subtype across 8 PCA datasets, as measured by the area under the receiver operating characteristic curve (AUC) using 100 MLs. **(b)** Unsupervised hierarchical clustering based on the 9 critical stemness subtype marker genes classified PCA patients from TCGA-PRAD into three subtypes (Predict Subtype). **(c)** K-M analysis showing the prognosis differences of the three stemness subtypes predicted by 9 genes. **(d)** Multi-class ROC analysis showed that the 9-gene predictor had excellent discriminative ability for the stemness subtype (AUC = 95.6%). **(e)** Radar graph displays the performance of the 9-gene stemness subtype predictor for predicting the malignancy across 3 PCA datasets, as measured by the area under the receiver operating characteristic curve (AUC) using 100 MLs. **(f)** Histogram shows the performances of the 9-gene predictor in predicting androgen deprivation therapy (ADT) response, metastasis and biochemical recurrence via 100 MLs. The top 15 MLs with the best performance are exhibited. **(g-i)** K-M analysis shows the effects of 9-gene-based stemness-related risk score (SRS) on DFI **(g)**, OS **(h)**, and DSS **(i)** of PCA patients from TCGA-PRAD. **(j)** Univariate COX analysis showed that SRS was one of risk factors for PCA patients. **(k-n)** ROC curves for predicting 1-, 3-, and 5-year PFI **(k)**, OS **(l)**, DFI **(m)**, and DSS **(n)** in patients from TCGA-PRAD. **(o)** ROC analysis showed that the predictive accuracy of SRS in PFI was superior to other clinical and molecular features in TCGA-PRAD. **(p-u)** K-M analysis shows the effects of SRS on prognosis of PCA patients from GSE21034 **(p)**, E-MTAB-6128 **(q)**, GSE46602 **(r)**, GSE84042 **(s)**, and prad_su2c_2019 **(t)**, and prostate_dkfz_2018 **(u)** datasets.

**Figure S12. Conservation of the three stemness subtypes in pan-tumors. (a, d, f, h)** Unsupervised hierarchical clustering based on the activity scores of the 18 stemness signatures classified pan-tumor patients from TcgaTargetGtex **(a)**, PCAWG **(d)**, ICGC **(f)**, and GSE2109 **(h)** into three subtypes. **(b, c)** K-M analysis shows distinct PFI **(b)**, OS, DFI, and DSS **(c)** of the three stemness subtypes from TcgaTargetGtex. **(e, g)** K-M analysis shows distinct OS of the three stemness subtypes from PCAWG **(e)** and ICGC **(g)**. **(i)** Dotplot shows the performance of the 9-gene stemness subtype predictor for predicting the HS subtype across 4 pan-tumor datasets, as measured by the AUC using 100 MLs. **(j)** Unsupervised hierarchical clustering based on the 9 critical stemness subtype marker genes classified pan-tumor patients into three subtypes (Predict Subtype). **(k, l)** K-M analysis shows distinct PFI **(k)**, OS, DFI, and DSS **(l)** of the three stemness subtypes predicted by 9 genes. **(m)** ROC curves for predicting 1-, 3-, and 5-year PFI, OS, DFI, and DSS in patients from TcgaTargetGtex. **(n)** K-M analysis shows the effects of SRS on PFI, OS, DFI, and DSS of pan-tumor patients from TcgaTargetGtex. **(o, p)** K-M analysis shows the effects of SRS on OS of pan-tumor patients from PCAWG **(o)** and ICGC **(p)**. **(q)** Univariate COX analysis showed that SRS was one of risk factors for pan-tumor patients. **(r)** Multivariate COX analysis showed that SRS was the most important independent risk factor for pan-tumor patients. **(s)** AUCs for predicting 1-, 3-, and 5-year PFI, OS, DFI, and DSS in patients from 33 tumor types.

**Figure S13. The impact of three pan-cancer stemness subtypes on ICB treatment and their predictive performance for ICB efficacy.** Related to Figure 9. **(a)** The K-M analysis shows that the differences in prognosis between HS patients and MS/LS patients are reduced after receiving ICB treatment. **(b)** Submap analysis reflects the sensitivity of the three pan-tumor stemness subtypes to an-PD-1, anti-PD-L1 and anti-CTLA-4 treatments. **(c-g)** Bladder cancer (BLCA, **c**), breast cancer (BRCA, **d**), non-small cell lung cancer (NSCLC, **e**), melanoma (SKCM, **f**), and clear cell renal cell carcinoma (ccRCC, **g**) are classified into three stemness subtypes based on 18 stemness signatures **(left)**. The response rate of each stemness subtype to ICB therapy (**medium**). The relationship between stemness subtypes and ICB efficacy (**right**). **(h)** Univariate logistic regression showing the effect of the three stemness subtypes on ICB efficacy. Separate analyzes are performed by cancer type. **(i, j)** Impacts of three TIDE subtypes and other predictive biomarkers on ICB efficacy, achieved by univariate **(i)** and multivariate **(j)** logistic regression analysis, respectively. **(k)** Nomogram for predicting non-response to ICB treatment based on logistic regression model. **(l)** Predictive receiver operating characteristic (ROC) curves of published predictive biomarkers and stemness subtypes for predicting the efficacy of ICB treatment. *p < 0.05, **p < 0.01, ***p < 0.001, ****p < 0.0001; ns, no significance.

**Figure S14. The impact of ICB treatment on stemness subtypes.** Related to Figure 9. **(a)** Unsupervised hierarchical clustering based on 18 stemness signatures classified the pan-tumor patients into three subtypes. These patients were treated with ICB with pre- and on/post-paired samples. **(b-d)** The proportion of three stemness subtypes before and on/after ICB treatment in all patients **(b)**, responders **(c)**, and non-responders **(d)**. **(e-g)** Paired samples of head and neck squamous cell carcinoma (HNSCC, **e**), esophageal adenocarcinoma (EAC, **f**), and SKCM **(g)** were divided into three subtypes based on 18 stemness signatures (**left**). The proportion of three stemness subtypes before and on/after ICB treatment in all patients, responders, and non-responders **(right)**.

### Additional Remarks

#### Remark A: Stemness scores are significantly associated with PCA clinicopathological and molecular features

As shown in **Figure S3k** and **Figure S3l**, mRNAsi was significantly positively correlated with PSA levels, androgen receptor (AR) expression, and tumor purity. mRNAsi was higher in pathological T4 stage (pT4) than in pT2 (p = 0.038) but was not significantly correlated with age, weight, or lymph node metastasis. Moreover, we found that mRNAsi was significantly positively correlated with TMB (p = 9e-06), somatic copy number alteration (SCNA), and fragment genomic alteration (FGA) but negatively correlated with methylation levels.

#### Remark B: Stemness score was negatively correlated with immune infiltration

Studies have shown that the tumor immune microenvironment (TIME) and stemness have a complex interaction, mutually regulating tumor development through different signaling pathways[57]. Therefore, we evaluated the relationship between the TIME and stemness. Forty-eight immune signature scores derived from published articles[58, 59] were quantified for each patient using the ssGSEA algorithm[60], and 553 PCA patients were clustered into two subtypes via consensus clustering[61]: "High immune infiltration" (High.immu, 252 samples, 45.6%) and " Low immune infiltration" subtype (Low.immu, 301 samples, 54.4%) (**Figure S4a**). Immune infiltration levels were also assessed using the ESTIMATE algorithm, which estimates the proportion of immune and stromal cells in the TME[62]. The results showed that the immune and stromal scores of the High.immu group were significantly higher than those of the Low.immu group (**Figure S4b**), indicating that High.immu contained abundant immune and stromal cells. Hypergeometric test and raincloud plot showed patients with high stemness tended to have lower immune infiltration levels, and vice versa (**Figure S4c**). Consistently, stemness scores were significantly negatively correlated with the immune and stromal scores (**Figure S4b, S4d)**. Using a novel deconvolution algorithm, DECEPTICON (https://github.com/Hao-Zou-lab/DECEPTICON), to assess the levels of immune cell infiltration, we found that the infiltration of CD4T, NK, and myeloid cells were significantly positively correlated with the mRNAsi scores (**Figure S4d**).

#### Remark C: Validation of three stemness subtypes

To verify the reproducibility and stability of our stemness classification results, we applied two different algorithms, non-negative matrix factorization (NMF)[63] and CensusClusterPlus[61], to classify the PCA samples (**Figures S6d-S6g**). Additionally, we used unsupervised hierarchical clustering to classify seven independent PCA bulk RNA-seq datasets[7, 9, 11-14, 16] to verify the generalizability of our clustering method (**Figures S6h-S6u**). The results demonstrated that, regardless of the clustering methods or datasets applied, patients could be divided into three subtypes. However, because of the small sample size of the West Coast Dream Team (WCDT) dataset[12], we were only able to divide the patients into three subtypes using the NMF algorithm (**Figure S6n**). This indicated the excellent stability and universality of our stemness classification method.

#### Remark D: HS enriches oncogenic signaling pathways

We performed gene set variation analysis[60] (GSVA), gene set enrichment analysis[64] (GSEA), and ingenuity pathway analysis (IPA) to investigate the functional annotations, signaling pathways, and underlying mechanisms associated with the PCA stemness subtypes. GSVA and GSEA revealed that LS mainly enriched nonspecific pathways (**Figure S8a, S8c**). In contrast, HS exhibited enrichment in cell cycle-related signaling pathways[65], which included cell cycle checkpoints, chromosome segregation, spindle assembly, centromere attachment, DNA replication, the telomerase pathway, MYC, and E2F targets (**Figure S8a, S8b**). GSVA showed that MS-enriched signaling pathways were similar to those of HS, but with slightly lower expression levels (**Figure S8a**). Additionally, GSEA revealed that MS significantly enriched the ribosome and translation-related pathways (**Figure** **S8d**). The advantage of IPA lies in the activity analysis of canonical pathways, which enables us to determine whether these pathways are activated or inhibited based on the expression or phosphorylation of significantly perturbed genes, proteins, or chemicals under the direction of literature findings. IPA revealed that pathways related to Intracellular and second messenger signaling, Cellular growth, proliferation and development, Cellular immune response, Cancer, and Cellular stress and injury were significantly inhibited in HS (**Figure** **S8e**). In contrast, pathways in Xenobiotic metabolism, Ingenuity toxicity list pathways, and Degradation/utilization/assimilation were activated (**Figure** **S8e**). This suggests that, although the cell cycle signaling pathway of HS is significantly overexpressed, its function remains inhibited. Notably, the activity status of the canonical pathways in MS was comparable to that in HS (**Figure S8g**), whereas LS displayed the opposite pattern (**Figure S8i**). Graphical summary demonstrated that phagocyte chemotaxis, granulocyte motility, and IL1 and estrogen receptor-related pathways were notably suppressed in the HS and MS subtypes (**Figure S8f, S8h**), which explains the observed lower levels of immune infiltration in these subtypes[66]. The opposite pattern holds for LS (**Figure S8j**). Additionally, heatmap showed that the pathways enriched in HS are closely linked to the development of many tumors, in contrast to LS. However, the role of MS appears to be ambiguous in different cancer types (**Figure S8k**).

#### Remark E

(1) Predictor can effectively distinguish stemness subtypes

Based on this 9-gene predictor, we used 100 MLs to predict the HS subtype repeatedly. We excluded the 10 models with the lowest AUCs and calculated the average AUCs for the following datasets: TCGA-PRAD (0.985 ± 0.020), GSE2103424 (0.943 ± 0.045), prostate_dkfz_2018 (0.978 ± 0.029), GSE70770 (0.991 ± 0.016), GSE94767 (0.985 ± 0.026), prad_su2c_2019 (0.897 ± 0.025), and WCDT (0.891 ± 0.060). The results are shown in **Figure S11a** and **Data S2**.

Furthermore, we used these nine genes to conduct unsupervised hierarchical clustering on 553 PCA samples, resulting in the identification of three distinct subtypes (**Figure S11b**). Notably, significant differences in the PFI were observed among these subtypes (p < 0.0001, **Figure S11c**). Multi-class ROC curve analysis demonstrated that the overall AUC for predicting the three subtypes was 95.6%, with AUC values of 99.1%, 92.7%, and 94.9% for predicting the HS, MS, and LS defined by the 18 gene sets, respectively (**Figure S11d**). These results suggest that our nine-gene predictor is highly effective in accurately distinguishing the stemness subtypes and can be used as an alternative clinically practical marker panel instead of the 18 gene sets.

(2) SRS can serve as a clinical prognostic biomarker

ROC analysis demonstrated that stemness-related risk score (SRS) can serve as a clinical prognostic biomarker with AUCs of 0.77, 0.71, and 0.64 for 1-year, 3-year, and 5-year PFI, respectively (**Figure S11k**). The AUCs for 1-year OS, 1-year DFI, and 1-year DSS were 0.99, 0.88, and 0.99, respectively (**Figures S11l-S11n**). Moreover, SRS was a better predictor of PFI than other clinical and molecular features (GS, PSA, pTNM stage, purity, etc.), as shown by the ROC curve (**Figure S11o**). To validate the prognostic value of SRS, we analyzed another six independent PCA cohorts[9, 11, 13, 15, 17, 19], and K-M analysis consistently demonstrated that the high SRS group had a significantly worse prognosis in all cohorts (**Figures S11p-S11u**).

#### Remark F

(1) The predictor is effective for distinguishing stemness subtypes in pan-tumors

We proceeded to test the 9-gene predictor's ability to identify the HS subtype in pan-tumors. We removed the 10 worst-performing MLs and calculated the mean AUCs for TcgaTargetGtex (10530 samples), PCAWG (1466 samples), GSE2109 (2158 samples), and ICGC (8746 samples) as 0.866 ± 0.03, 0.994 ± 0.004, 0.980 ± 0.02, and 0.849 ± 0.017, respectively (**Figure S12i, Data S2**). We further applied unsupervised clustering to the TcgaTargetGtex dataset based on these 9 genes (**Figure S12j**). Significant differences in PFI, OS, DFI, and DSS were observed among the three identified subtypes (**Figures S12k, S12l**).

(2) SRS can predict the clinical prognosis of pan-tumor patients

Given the commonality of the stemness subtypes, we investigated the performance of SRS in predicting the prognosis of pan-tumors. ROC curve revealed that SRS had an AUC of 0.62, 0.62, 0.60, and 0.64 for 5-year PFI, OS, PFI, and DSS, respectively (**Figure S12m**). K-M analysis showed that patients with low SRS had significantly better PFI, OS, DFI, and DSS than those with high SRS (**Figure S12n**). This trend was consistent across both the PACWG and ICGA datasets (**Figures S12o, S12p**). Univariate COX analysis identified SRS, age, male sex, purity, and pTNM stage as risk factors, whereas female sex was protective in pan-tumors (**Figure S12q**). Furthermore, multivariate COX analysis demonstrated that SRS was the most significant independent risk factor for pan-tumors (**Figure S12r**, HR = 2.27, p = 8.3e-4). We also tested SRS in predicting 1-year, 3-year, and 5-year PFI, OS, DFI, and DSS for 33 tumor types in TCGA and found that it performed well for MESO, KICH, ACC, KIRP, and PRAD (**Figure S12s**, dashed line: AUC = 0.7).

#### Remark G

(1) HS patients are more inclined to benefit from immunotherapy

K-M analysis showed that the prognosis of HS subtype was improved after ICB treatment, suggesting that ICB conferred a similar survival benefit to the HS and MS subtypes as the LS subtype (**Figure S13a**). Submap analysis[67] indicated that HS was most strongly associated with anti-PD-L1 response, while LS was likely to be associated with anti-PD-L1 resistance. In contrast, MS showed a mixed association with both response and resistance to anti-PD1, reflecting its transitional status (**Figure S13b**). Separate clustering analysis of each cancer type (sample size >50) showed that these tumors could be divided into three subtypes based on 18 stemness signatures. In UC, BRCA, NSCLC, and SKCM, HS had a significantly higher response rate than LS and was associated with response, while LS was associated with non-response (**Figures S13c-S13f**). However, in ccRCC, the response rates of the three subtypes did not differ significantly (**Figure S13g**), which may reflect the unique immune therapeutic response mechanism of ccRCC[68, 69].

(2) Stemness subtype was an effective predictor of ICB responsiveness

We further compared the stemness subtypes with other ICB predictive biomarkers using the IMvigor210 dataset. Univariate logistic regression analysis revealed that the LS subtype and infiltrated immune phenotype posed a higher risk for ICB response, whereas TMB, neoantigen load, IC2, and genomically unstable subtype acted as protective factors for response (**Figure S13i**). Furthermore, our multivariate logistic analysis demonstrated that LS was an independent risk factor for ICB response (**Figure S13j**, log odds = 1.67). In addition, the nomogram indicated that LS contributed the most to ICB resistance (**Figure S13k**). The predictive performance of the stemness subtype for ICB efficacy was further evaluated using receiver operating characteristic (ROC) curves and compared with published biomarkers. The results demonstrated that both the stemness subtype and SRS were effective predictors of ICB efficacy (**Figure S13l**, p = 0.0021 and 0.0098, respectively), with their predictive performance surpassing that of most biomarkers (**Figure S13l**).

### Methods S1

#### Patients and datasets collection

Single-cell RNA-seq datasets of PCA were collected from the Gene Expression Omnibus (GEO) and Prostate Cell Atlas[4]. We extracted 75350 prostate epithelial cells from 90 samples across the five datasets. Bulk RNA-seq datasets of PCA were collected from various sources, including TCGA, UCSC Xena, GEO, cBioPortal, ArrayExpress[70], and the WCDT[12]. Among them, seven datasets (a total of 1641 samples) including tumor and benign samples were used for stemness evaluation and differential expression analysis, and eight datasets (a total of 1912 samples) including follow-up information were used for classification and subtype evaluation. Follow-up information for TCGA-PRAD was obtained from the UCSC Xena. The DNA methylation, CAN, and somatic mutation data based on the whole-exome sequencing platform were downloaded from TCGA using the TCGAbiolinks package (v2.25.3). The gene expression matrix for PSC was collected from the Progenitor Cell Biology Consortium (PCBC) using the synapser package (v0.15.35). Four pan-cancer cohorts were collected from UCSC Xena and GEO, with a total of 28,381 samples. The pretreatment bulk RNA-seq cohort of patients who received ICB treatment was obtained from various databases, including GEO, ArrayExpress, the Database of Genotypes and Phenotypes (dbGaP), the National Center for Biotechnology Information (NCBI), the European Genome-Phenome Archive (EGA), and supplements or R packages provided by the authors. This cohort included 2641 samples from 12 tumor types in 36 datasets. Details and sources for all datasets are listed in **Table S1**.

#### Study Design

The design of this study is as follows:

(1) Evaluation of stemness and its relationship with clinical and molecular features of prostate cancer (PCA): First, we used the cytoTRACE algorithm[71] to assess the stemness scores (cytoTRACE score) of epithelial cells in each single-cell RNA-seq (scRNA-seq) dataset and compared the differences in cytoTRACE scores between normal and tumor cells, as well as between low-grade (Gleason score [GS] ≤ 7) and high-grade (GS > 7) PCA cells. Second, we used the OCLR machine learning (ML) algorithm[72] to evaluate the stemness indices (mRNAsi, mDNAsi, EREG_mDNAsi, DMPsi) of samples in 7 PCA bulk RNA-seq datasets and 1 DNA methylation dataset. Then, we compared the differences in these stemness indices between benign and tumor samples, as well as between low-grade and high-grade PCA samples. Finally, we explored the association between mRNAsi and other clinical (such as pathological TNM staging, age, weight, serum prostate specific antigen [PSA]) and molecular features (such as tumor mutational burden [TMB], somatic copy number alterations) of PCA.

(2) PCA stemness classification: First, we identified 495 stemness marker genes through gene differential expression (DE) analysis and correlation analysis. Then, we investigated gene sets of these genes on MSigDB[73], and obtained 18 hazardous gene sets significantly associated with prognosis through univariate COX analysis. Finally, we divided PCA into three subtypes using unsupervised hierarchical clustering based on these 18 stemness signatures.

(3) Characterization of clinical and molecular features of stemness subtypes: First, we used two additional algorithms - Consensus Clustering[61] and Non-negative Matrix Factorization (NMF)[63] - based on 18 stemness signatures to classify PCA, to test the stability of the subtypes. Next, we characterized the differences in clinical and pathological features, molecular features, tumor immune microenvironment (TIME), functional annotations and signaling pathways, as well as drug sensitivity of three stemness subtypes.

(4) Construction and validation of the stemness subtype predictor: Firstly, we performed weighted gene co-expression network analysis (WGCNA)[74] on 8 PCA bulk RNA-seq datasets to identify hub genes of the HS subtype. Then, we obtained 40 genes by intersecting these hub genes with 495 stemness marker genes. The stemness subtype predictor was jointly developed using 4 PCA datasets and 76 MLs, consisting of 9 genes in total. Subsequently, we collected 60 real-world PCA samples to validate the expression differences of these 9 genes in benign samples, low-grade and high-grade PCA samples. Finally, we validated the ability of this predictor to predict the stemness subtype. In addition, we also validated the predictive performance of this predictor for other clinical events, such as benign and malignant differentiation, androgen deprivation therapy (ADT) efficacy, prognosis, biochemical recurrence, progression, and metastasis.

(5) Analysis of the pan-tumor landscape of the stemness subtypes: First, we performed unsupervised hierarchical clustering on four pan-tumor datasets based on 18 stemness signatures, dividing each dataset into three stemness subtypes. Next, we validated the sensitivity differences of the three stemness subtypes to immune checkpoint blockade (ICB) therapy using a bulk RNA-seq pan-tumor cohort of baseline samples treated with ICB. Finally, we tested the performance of the 9-gene stemness subtype predictor in pan-tumors.

#### Single-cell RNA-seq data processing and identification of epithelial cells

In the prostate_portal_300921 dataset[4], epithelial cells that were annotated by the original authors were directly extracted. The GSE193337[1], GSE185344[2], GSE176031[3], and GSE141445[5] datasets were processed using uniform methods and standards with the Seurat package[75] (v4.2.1). Cells with less than 200 genes or more than 20% mitochondrial genes were removed, as well as features detected in less than 3 cells. Normalization was performed using Seurat’s defaults and 2000 hypervariable features were selected for downstream analysis. The sample integration and batch effect removal were performed using the anchor-based canonical correlation analysis (CCA) method, followed by data normalization using the ScaleData function. The integrated RNA assay was used for dimensionality reduction and clustering, and principal component analysis (PCA) was performed using the RunPCA function. Louvain clustering was done with 30 PCs and resolution = 1, and the results were visualized in two-dimensional space using the uniform manifold approximation and projection (UMAP). To identify marker genes for each cluster, differential expression (DE) analysis was conducted using the FindAllMarkers function. SingleR package[76] (v2.0.0) was used to annotate all clusters with relevant marker genes reported in the literature. Finally, epithelial cells were extracted for subsequent stemness analysis.

#### Differential expression (DE) analysis

For scRNA-seq data, the FindAllMarkers function (min.pct = 0.1, logfc.threshold = 0.25) was used to identify marker genes for each Louvain cluster. DE analysis was performed on tumor and benign epithelial cells, as well as high-grade (GS > 7) and low-grade (GS ≤ 7) epithelial cells, using the default settings of the scCODE package[77] (v1.2.0.1, https://github.com/XZouProjects/scCODE). For bulk RNA-seq datasets without negative values, DE analysis was also performed using scCODE[77]. For GSE35988[6] and GSE6099[10] with negative values, DE analysis was performed using the generalized linear model method of the Limma package[78]. In all DE analyses, genes with Bonferroni FDR-corrected p-values < 0.05 were considered DE genes based on the two-sided Wilcoxon rank-sum test, but genes with Detected_Times < 3 in scCODE results were excluded.

#### Bulk RNA-seq data processing

A log2 transformation of the counts, TPM, and FPKM expression matrices was performed. Use the liftOver tool to transform the genome version if necessary. Additionally, the removeBatchEffect function of the Limma package[78] (v3.52.4) was used to integrate datasets.

#### Stemness analysis

For single-cell RNA-seq data, the cytoTRACE package[71] (v0.3.3) was used to assess the stemness of prostate epithelial cells. The package is based on the hypothesis that "cells with higher differentiation have more complex transcriptomes" and can evaluate the differentiation potential of single-cell subpopulations. The higher cytoTRACE score, which reflects the higher gene counts in a single cell, indicates lower cell differentiation and higher stemness level[71]. For bulk RNA-seq and DNA methylation data, the One Class Linear Regression (OCLR) ML algorithm[72] was used to quantify the stemness of samples. By constructing prediction models using the mRNAsi probe with 12,945 genes, we calculated the stemness index based on transcriptome. Similarly, by constructing prediction models based on the mDNAsi probe containing 219 methylated regions, the EREG_mDNAsi probe with 87 methylated regions, and the DMPsi probe containing 62 methylated regions, we calculated the stemness indexes based on DNA methylation data, namely mDNAsi, EREG_mDNAsi, and DMPsi. The higher the scores of these indexes, the higher the stemness of the samples[72].

In the K-M analysis, the optimal cut-point was calculated via the built-in surv_cutpoint function in the survminer package (v0.4.9) to divide patients into high stemness and low stemness groups.

#### Pathway analysis

Download the reference gene-sets for GSEA analysis, including GOBP, Hallmark, KEGG, and Reactome, from the Molecular Signatures Database[73] (MSigDB, http://www.gsea-msigdb.org/gsea/index.jsp). The Investigate Gene Sets tool (http://www.gsea-msigdb.org/gsea/msigdb/annotate.jsp) can be used to identify the overlapping gene-sets of the submitted gene-lists in MSigDB.

Use the "ssgsea" method of the GSVA package[60] (v1.44.5) to perform single-sample gene set enrichment analysis (ssGSEA) and assign corresponding signature activity scores to each sample.

Evaluate the enrichment scores of signaling pathways using gene set variation analysis (GSVA) with the "gsva" method of the GSVA package[60]. Use the Limma package[78] to perform differential analysis on the enrichment scores, which can identify pathways significantly enriched in different stemness subtypes.

Perform gene set enrichment analysis (GSEA) on pre-ranked DE gene-lists using the clusterProfiler package[64] (v4.4.4), with reference to the GOBP, Hallmark, KEGG, and Reactome gene-sets.

Use the QIAGEN IPA software (IPA Winter Release December 2022, https://digitalinsights.qiagen.com/products-overview/discovery-insights-portfolio/analysis-and-visualization/qiagen-ipa/) to perform ingenuity pathway analysis (IPA) on identified DE genes of each stemness subtype. The Pathways tool can expose the activation or inhibition status of canonical pathways in each subtype. The Disease and Function tool can reveal the correlation between the interested gene sets and biological functions or diseases.

#### Clustering Analysis

Hierarchical clustering is a widely used unsupervised clustering technique that partitions data into a hierarchical structure of nested clusters. The hclust function in R software is a popular implementation of this method. First, we applied hclust to the normalized data using default parameters, which perform agglomerative clustering using the complete linkage method. We then visualized the results using a dendrogram, which represents the hierarchical structure of the clusters. To specify a desired number of clusters, we used the cutree function to cut the dendrogram. Additionally, we performed PCA using the prcomp function, and the resulting samples were projected into three-dimensional space using the scatterplot3d package (v0.3.42).

To ensure the accuracy of our classification results, we employed two additional techniques: consensus clustering and non-negative matrix factorization (NMF). Consensus clustering was carried out using the ConsensusClusterPlus package[61] (v1.58.0). To improve the reliability of our clustering, we repeated the process 1000 times, randomly selecting 80% of the samples each time. Meanwhile, we employed the NMF algorithm from the NMF package[63] (v0.24.0), which decomposes the matrix and performs 100 repetitions for stable, unsupervised clustering.

The optimal number of clusters was comprehensively determined by the consensus heatmap, the cumulative distribution function (CDF) curves, and the proportion of ambiguous clustering algorithm (PAC)[79] (**Figure S6a**).

#### Weighted gene co-expression network analysis (WGCNA)

We used the WGCNA package[74] (v1.71) to construct the gene co-expression network and identified modules and hub genes that were most strongly correlated with HS. First, we normalized the gene expression data and constructed an adjacency matrix by filtering out the outlier samples. We then selected an appropriate soft threshold (β) to ensure that the network conformed to a scale-free distribution and calculated the topological overlap matrix (TOM). Next, we used dynamic tree cutting to identify modules and represented each module's expression profile using a module eigengene (ME). Finally, we calculated the correlation between each module and HS and identified the module with the highest correlation as the HS module. Within the HS module, genes meeting the criteria of GS > 0.4 & MS > 0.4 & p < 0.05 were considered hub genes (**Figure 5a**).

#### Protein–protein interaction network analysis (PPI)

We constructed a PPI network of Core.Sig (**Figure S10a**) using the STRING database[80], and the network was clustered to a specified "MCL inflation parameter". Genes without interactions were omitted.

#### Somatic mutation and copy number variation analysis

To explore the genomic characteristics and mutation spectrum of the stemness subtype, we conducted somatic mutation and copy number variation analyses. We began by downloading somatic mutation data and copy number variation data of PCA from TCGA. Using GISTIC_2.0 (v6.15.30, <https://cloud.genepattern.org/gp/pages/index.jsf>), we identified significant amplified or deleted genomic regions across the entire genome, and calculated the G-score for each region, taking into account the amplitude and frequency of variations. We used maftools[81] (v2.12.0) and RCircos[82] (v1.2.2) packages to analyze and visualize the mutation types, frequencies, and copy number variations.

#### Evaluation of immune patterns

To evaluate the immune patterns of the PCA samples, we collected 48 immune-related signatures published by He et al.[58] and Bagaev et al.[59] and used the ssGSEA algorithm[60] to calculate the active scores of these immune-related features. Based on these ssGSEA scores, PCA samples were classified into different immune subtypes using the consensus clustering method[61]. We also evaluated the tumor microenvironment (TME) using the ESTIMATE package[62] (v1.0.13). This package can be used to estimate the levels of stromal cells, immune infiltration, and tumor purity in tumor tissues based on transcriptome data. In addition, we assessed the abundance of immunocyte infiltrating in tissue samples using the CIBERSORT algorithm[83] and our team's newly developed deconvolution algorithm, DECEPTICON (v1.0, <https://github.com/Hao-Zou-lab/DECEPTICON>).

#### Evaluation of immunotherapy efficacy

To evaluate the responsiveness of different stemness subtypes to immunotherapy, we employed two algorithms: the Tumor Immune Dysfunction and Exclusion[84] (TIDE, http://tide.dfci.harvard.edu/) and unsupervised subclass mapping[67] (SubMap, v4.0, https://cloud.genepattern.org/gp/pages/index.jsf). We then validated the results by analyzing pretreatment bulk RNA-seq data from pan-tumor samples that received ICB. We also validated the effect of immunotherapy on stemness using pre- and on/post-ICB paired samples from the same individuals.

The TIDE algorithm evaluates two scores - the dysfunction score and exclusion score - based on two mechanisms of tumor immune escape: T cell dysfunction and T cell exclusion. By analyzing gene expression data, the algorithm can predict the potential for tumor immune escape and the response to immunotherapy[84]. We used the TIDE package (v1.3) in Python software to predict patient responsiveness to immunotherapy. To increase the sensitivity of responder prediction, we set the threshold for distinguishing responders from non-responders to -0.2 (vthres = -0.2).

We also utilized Submap, an unsupervised machine learning method, to measure the similarity between stemness subtypes and the efficacy of immunotherapy. Submap can identify common subtypes in two independent datasets[67], which helped us to determine the sensitivity of different subtypes to immunotherapy.

#### Evaluation of drug sensitivity

Based on the Genomics of Drug Sensitivity in Cancer[85] (GDSC v8.4, <https://www.cancerrxgene.org/>) and Cancer Therapeutics Response Portal[86] (CTRP v2, <https://portals.broadinstitute.org/ctrp/>) databases, we used the oncoPredict package[87] (v0.2) to evaluate the sensitivity of different stemness subtypes to drugs. The sensitivity was measured using the half-maximal inhibitory concentration (IC50). This method uses gene expression profiles and drug sensitivity data from a large number of cancer cell lines to construct predictive models. The IC50 of drugs in PCA patients was evaluated using ridge regression algorithm.

#### Identification of stemness marker genes and developing of signatures for stemness classifications

The identification process is shown in **Figure S5a**.

Identification of stemness marker genes based on scRNA-seq data (scStem.Sig). We first identified stemness marker genes in malignant epithelial cells (Tumor scStem.Sig) by analyzing GSE193337[1], GSE185344[2], GSE176031[3], and prostate_portal_300921[4] datasets, which included both tumor and benign cells. To identify genes that were significantly positively correlated with cytoTRACE scores (cytoGenes, R > 0 and FDR < 1e-05), we performed Spearman correlation analysis between the gene expression levels and cytoTRACE scores of malignant epithelial cells in the four datasets. Meanwhile, DE analysis was performed on malignant and benign epithelial cells, and selected significantly up-regulated DE genes in malignant epithelial cells (Tumor.DE, Detected_times ≥ 3 & FDR < 0.05 & FC > 0). To identify genes that are up-regulated in malignant epithelial cells and positively correlated with stemness, we first took the intersection of cytoGenes and Tumor.DE to obtain cytoTumor.DE for each dataset. The arithmetic mean of Spearman R for each gene in the four datasets was calculated. Finally, genes with an average R > 0.4 were merged into Tumor scStem.Sig. We then used two datasets (GSE141445[5] and GSE176031[3]) with Gleason Score (GS) information to identify stemness marker genes in high-grade PCA epithelial cells. To do this, we applied the same method as described above and used Spearman correlation analysis to identify cytoGenes. We performed DE analysis on both high-grade (GS > 7) and low-grade (GS ≤ 7) PCA epithelial cells to identify significantly upregulated DE genes in high-grade PCA (High.DE, Detected_times ≥ 3 & FDR < 0.05 & FC > 0). The intersection of cytoGenes and High.DE resulted in cytoHigh.DE for each dataset. Finally, we considered genes with an average Spearman R value > 0.4 in these two datasets as High scStem.Sig. We ultimately obtained scStem.Sig (288 genes) by taking the intersection of Tumor scStem.Sig and High scStem.Sig (**Figure 2a, left**).

Identification of stemness marker genes based on bulk RNA-seq data (Bulk Stem.Sig). The principle is the same as above. We performed Spearman correlation analysis between the gene expression levels and mRNAsi of the samples in 7 bulk RNA-seq datasets[6-10], selecting genes that were significantly positively correlated with mRNAsi (mRNAsi.Genes, R > 0 & FDR < 1e-05). We also conducted DE analysis on PCA and benign prostate samples, selecting genes that were significantly upregulated in tumors (Tumor.DE, Detected_times ≥ 3 & FDR < 0.05 & FC > 0). To obtain genes that were positively correlated with mRNAsi and significantly upregulated in tumors, we took the intersection of mRNAsi.Genes and Tumor.DE, resulting in mRNAsi.Tumor.DE for each dataset. We calculated the arithmetic mean of Spearman R for each gene in the 7 datasets. Finally, we combined genes with an average value greater than 0.4 into Bulk Stem.Sig (**Figure 2a, right**).

By combining scStem.Sig and Bulk Stem.Sig, we obtained stemness marker genes (Stem.Sig), which comprised 495 genes. We then used MSigDB[73] to investigate the top 10 gene sets that overlapped with GOBP, HALLMARK, KEGG, and REACTOME, and evaluated their expression scores using the ssGSEA algorithm[60]. Subsequently, we conducted univariate COX analysis and found that 27 pathways were significantly correlated with PFI (p < 0.05). After adjusting the p values using the Benjamini–Hochberg method, we identified 20 significant pathways (p.adjusted ≤ 0.05), of which two were protective factors and 18 were risk factors (**Figure S5b**). Finally, we selected the18 significant risk pathways for subsequent stemness classification.

#### Construction and verification of stemness subtype predictor

Currently, there are many machine learning algorithms (MLs), and different MLs may have different adaptability and advantages for different data types and problems. Therefore, combining multiple algorithms can improve the model's generalization ability and accuracy. Similarly, different datasets have different features and noise, and combining multiple datasets can provide more information and features, which can help enhance the model's expressive power and robustness and avoid overfitting or underfitting problems. Using multiple algorithms and datasets for joint development can also help determine which factors are most important for the predictor's performance. This helps to better understand the problem itself and provide better guidance for further improving the predictor. Therefore, choosing multiple MLs and multiple datasets for joint construction of predictor is an effective method to improve the accuracy and robustness. To develop an accurate prediction model with exceptional generalization ability, we employed a joint analysis approach using multiple MLs and datasets. See **Figure S10a** for detailed procedures.

The predictor generation and validation procedure was as follows: (a) We started by performing WGCNA on eight datasets respectively (**Figure 5a, Figures S10b-S10g**), which yielded 531 hub genes (Hub.Sig). These genes were intersected with 495 Stem.Sig genes, resulting in 40 genes (Core.Sig). (b) Univariate COX analysis showed that these 40 genes were all prognostic genes of TCGA-PRAD. (c) After removing genes with no interactions via PPI, 36 genes remained (PPI.Sig, **Figure 5b**). (d) Next, we extensively collected 100 MLs based on caret package (v6.0-93), as listed in **Data S3**, and selected 76 algorithms that could be used to construct models based on a single gene to develop stemness subtype predictors of the 36 genes. These predictors were optimized using 5-fold cross-validation and 10 repetitions of optimization. Each gene could construct 76 single-gene prediction models for predicting the HS subtype, and we evaluated their performance using the area under the receiver operating characteristic curve (AUC). (e) Subsequently, we averaged the AUCs of the 76 single-gene prediction models constructed by the same gene, and candidate genes for the predictor were selected based on an average AUC > 0.9. (f) We applied this process to four datasets, TCGA-PRAD, GSE21034[9], prostate_dkfz_2018[13], and GSE70770 datasets[14], respectively, and obtained the intersection of the four candidate gene panels. Finally, the 9 genes generated by the intersection were selected as the stemness subtype predictor (**Figure 5c**).

To assess the accuracy and robustness of our predictor, we validated it using 100 MLs across 8 PCA and 4 pan-cancer bulk RNA-seq datasets. Additionally, we tested the predictor's clinical utility by evaluating its ability to distinguish between benign and malignant tumors, predict androgen deprivation therapy (ADT) efficacy, biochemical recurrence, metastasis, disease progression, and prognosis. The predictive performance was measured by AUC. Multi-classification ROC curve analysis was performed by the confusionMatrix package (v0.3.6).

#### Calculation of SRS

First, univariate and multivariate COX analyses were performed on the nine predictor genes. The regression coefficient (β) of each covariate was obtained using multivariate COX analysis. SRS was constructed using the weighted estimated multivariate COX regression coefficients. The formula is:

SRS = -0.525*TOP2A_expr_ + 0.847*TPX2_expr_ + 0.043*CDK1_expr_ + 1.101*KIF4A_expr_ + 0.758*BUB1_expr_ - 0.36*HMMR_expr_ - 0.664*DLGAP5_expr_ - 0.517*NCAPG_expr_ - 0.195*SKA3_expr_.

In the K-M analysis, the best cutoff point can be calculated using the built-in surv_cutpoint function in the Survminer R package to divide patients into high and low SRS groups.

### Additional References

1.	Heidegger I, Fotakis G, Offermann A, Goveia J, Daum S, Salcher S, Noureen A, Timmer-Bosscha H, Schäfer G, Walenkamp A *et al*: **Comprehensive characterization of the prostate tumor microenvironment identifies CXCR4/CXCL12 crosstalk as a novel antiangiogenic therapeutic target in prostate cancer**. *Mol Cancer* 2022, **21**(1):132.

2.	Wong HY, Sheng Q, Hesterberg AB, Croessmann S, Rios BL, Giri K, Jackson J, Miranda AX, Watkins E, Schaffer KR *et al*: **Single cell analysis of cribriform prostate cancer reveals cell intrinsic and tumor microenvironmental pathways of aggressive disease**. *Nat Commun* 2022, **13**(1):6036.

3.	Song H, Weinstein HNW, Allegakoen P, Wadsworth MH, 2nd, Xie J, Yang H, Castro EA, Lu KL, Stohr BA, Feng FY *et al*: **Single-cell analysis of human primary prostate cancer reveals the heterogeneity of tumor-associated epithelial cell states**. *Nat Commun* 2022, **13**(1):141.

4.	Tuong ZK, Loudon KW, Berry B, Richoz N, Jones J, Tan X, Nguyen Q, George A, Hori S, Field S *et al*: **Resolving the immune landscape of human prostate at a single-cell level in health and cancer**. *Cell Rep* 2021, **37**(12):110132.

5.	Chen S, Zhu G, Yang Y, Wang F, Xiao YT, Zhang N, Bian X, Zhu Y, Yu Y, Liu F *et al*: **Single-cell analysis reveals transcriptomic remodellings in distinct cell types that contribute to human prostate cancer progression**. *Nat Cell Biol* 2021, **23**(1):87-98.

6.	Grasso CS, Wu YM, Robinson DR, Cao X, Dhanasekaran SM, Khan AP, Quist MJ, Jing X, Lonigro RJ, Brenner JC *et al*: **The mutational landscape of lethal castration-resistant prostate cancer**. *Nature* 2012, **487**(7406):239-243.

7.	Luca BA, Brewer DS, Edwards DR, Edwards S, Whitaker HC, Merson S, Dennis N, Cooper RA, Hazell S, Warren AY *et al*: **DESNT: A Poor Prognosis Category of Human Prostate Cancer**. *Eur Urol Focus* 2018, **4**(6):842-850.

8.	Friedrich M, Wiedemann K, Reiche K, Puppel SH, Pfeifer G, Zipfel I, Binder S, Köhl U, Müller GA, Engeland K *et al*: **The Role of lncRNAs TAPIR-1 and -2 as Diagnostic Markers and Potential Therapeutic Targets in Prostate Cancer**. *Cancers (Basel)* 2020, **12**(5).

9.	Taylor BS, Schultz N, Hieronymus H, Gopalan A, Xiao Y, Carver BS, Arora VK, Kaushik P, Cerami E, Reva B *et al*: **Integrative genomic profiling of human prostate cancer**. *Cancer Cell* 2010, **18**(1):11-22.

10.	Tomlins SA, Mehra R, Rhodes DR, Cao X, Wang L, Dhanasekaran SM, Kalyana-Sundaram S, Wei JT, Rubin MA, Pienta KJ *et al*: **Integrative molecular concept modeling of prostate cancer progression**. *Nat Genet* 2007, **39**(1):41-51.

11.	Abida W, Cyrta J, Heller G, Prandi D, Armenia J, Coleman I, Cieslik M, Benelli M, Robinson D, Van Allen EM *et al*: **Genomic correlates of clinical outcome in advanced prostate cancer**. *Proc Natl Acad Sci U S A* 2019, **116**(23):11428-11436.

12.	Quigley DA, Dang HX, Zhao SG, Lloyd P, Aggarwal R, Alumkal JJ, Foye A, Kothari V, Perry MD, Bailey AM *et al*: **Genomic Hallmarks and Structural Variation in Metastatic Prostate Cancer**. *Cell* 2018, **174**(3):758-769.e759.

13.	Gerhauser C, Favero F, Risch T, Simon R, Feuerbach L, Assenov Y, Heckmann D, Sidiropoulos N, Waszak SM, Hübschmann D *et al*: **Molecular Evolution of Early-Onset Prostate Cancer Identifies Molecular Risk Markers and Clinical Trajectories**. *Cancer Cell* 2018, **34**(6):996-1011.e1018.

14.	Ross-Adams H, Lamb AD, Dunning MJ, Halim S, Lindberg J, Massie CM, Egevad LA, Russell R, Ramos-Montoya A, Vowler SL *et al*: **Integration of copy number and transcriptomics provides risk stratification in prostate cancer: A discovery and validation cohort study**. *EBioMedicine* 2015, **2**(9):1133-1144.

15.	Mortensen MM, Høyer S, Lynnerup AS, Ørntoft TF, Sørensen KD, Borre M, Dyrskjøt L: **Expression profiling of prostate cancer tissue delineates genes associated with recurrence after prostatectomy**. *Sci Rep* 2015, **5**:16018.

16.	Long Q, Xu J, Osunkoya AO, Sannigrahi S, Johnson BA, Zhou W, Gillespie T, Park JY, Nam RK, Sugar L *et al*: **Global transcriptome analysis of formalin-fixed prostate cancer specimens identifies biomarkers of disease recurrence**. *Cancer Res* 2014, **74**(12):3228-3237.

17.	Fraser M, Sabelnykova VY, Yamaguchi TN, Heisler LE, Livingstone J, Huang V, Shiah YJ, Yousif F, Lin X, Masella AP *et al*: **Genomic hallmarks of localized, non-indolent prostate cancer**. *Nature* 2017, **541**(7637):359-364.

18.	Jain S, Lyons CA, Walker SM, McQuaid S, Hynes SO, Mitchell DM, Pang B, Logan GE, McCavigan AM, O'Rourke D *et al*: **Validation of a Metastatic Assay using biopsies to improve risk stratification in patients with prostate cancer treated with radical radiation therapy**. *Ann Oncol* 2018, **29**(1):215-222.

19.	Nabila E: **Expression array for Multi-omics molecular profiling of primary prostate adenocarcinoma**. 2018.

20.	Alumkal JJ, Sun D, Lu E, Beer TM, Thomas GV, Latour E, Aggarwal R, Cetnar J, Ryan CJ, Tabatabaei S *et al*: **Transcriptional profiling identifies an androgen receptor activity-low, stemness program associated with enzalutamide resistance**. *Proc Natl Acad Sci U S A* 2020, **117**(22):12315-12323.

21.	Tamura K, Furihata M, Tsunoda T, Ashida S, Takata R, Obara W, Yoshioka H, Daigo Y, Nasu Y, Kumon H *et al*: **Molecular features of hormone-refractory prostate cancer cells by genome-wide gene expression profiles**. *Cancer Res* 2007, **67**(11):5117-5125.

22.	Sharma NL, Massie CE, Ramos-Montoya A, Zecchini V, Scott HE, Lamb AD, MacArthur S, Stark R, Warren AY, Mills IG *et al*: **The androgen receptor induces a distinct transcriptional program in castration-resistant prostate cancer in man**. *Cancer Cell* 2013, **23**(1):35-47.

23.	Erho N, Crisan A, Vergara IA, Mitra AP, Ghadessi M, Buerki C, Bergstralh EJ, Kollmeyer T, Fink S, Haddad Z *et al*: **Discovery and validation of a prostate cancer genomic classifier that predicts early metastasis following radical prostatectomy**. *PLoS One* 2013, **8**(6):e66855.

24.	Mariathasan S, Turley SJ, Nickles D, Castiglioni A, Yuen K, Wang Y, Kadel EE, III, Koeppen H, Astarita JL, Cubas R *et al*: **TGFβ attenuates tumour response to PD-L1 blockade by contributing to exclusion of T cells**. *Nature* 2018, **554**(7693):544-548.

25.	Snyder A, Nathanson T, Funt SA, Ahuja A, Buros Novik J, Hellmann MD, Chang E, Aksoy BA, Al-Ahmadie H, Yusko E *et al*: **Contribution of systemic and somatic factors to clinical response and resistance to PD-L1 blockade in urothelial cancer: An exploratory multi-omic analysis**. *PLoS Med* 2017, **14**(5):e1002309.

26.	Pusztai L, Yau C, Wolf DM, Han HS, Du L, Wallace AM, String-Reasor E, Boughey JC, Chien AJ, Elias AD *et al*: **Durvalumab with olaparib and paclitaxel for high-risk HER2-negative stage II/III breast cancer: Results from the adaptively randomized I-SPY2 trial**. *Cancer Cell* 2021, **39**(7):989-998.e985.

27.	Wolf DM, Yau C, Wulfkuhle J, Brown-Swigart L, Gallagher RI, Lee PRE, Zhu Z, Magbanua MJ, Sayaman R, O'Grady N *et al*: **Redefining breast cancer subtypes to guide treatment prioritization and maximize response: Predictive biomarkers across 10 cancer therapies**. *Cancer Cell* 2022, **40**(6):609-623.e606.

28.	Keenan TE, Guerriero JL, Barroso-Sousa R, Li T, O'Meara T, Giobbie-Hurder A, Tayob N, Hu J, Severgnini M, Agudo J *et al*: **Molecular correlates of response to eribulin and pembrolizumab in hormone receptor-positive metastatic breast cancer**. *Nat Commun* 2021, **12**(1):5563.

29.	Choueiri TK, Fishman MN, Escudier B, McDermott DF, Drake CG, Kluger H, Stadler WM, Perez-Gracia JL, McNeel DG, Curti B *et al*: **Immunomodulatory Activity of Nivolumab in Metastatic Renal Cell Carcinoma**. *Clin Cancer Res* 2016, **22**(22):5461-5471.

30.	Braun DA, Hou Y, Bakouny Z, Ficial M, Sant' Angelo M, Forman J, Ross-Macdonald P, Berger AC, Jegede OA, Elagina L *et al*: **Interplay of somatic alterations and immune infiltration modulates response to PD-1 blockade in advanced clear cell renal cell carcinoma**. *Nat Med* 2020, **26**(6):909-918.

31.	Motzer RJ, Rini BI, McDermott DF, Redman BG, Kuzel TM, Harrison MR, Vaishampayan UN, Drabkin HA, George S, Logan TF *et al*: **Nivolumab for Metastatic Renal Cell Carcinoma: Results of a Randomized Phase II Trial**. *J Clin Oncol* 2015, **33**(13):1430-1437.

32.	Motzer RJ, Tannir NM, McDermott DF, Arén Frontera O, Melichar B, Choueiri TK, Plimack ER, Barthélémy P, Porta C, George S *et al*: **Nivolumab plus Ipilimumab versus Sunitinib in Advanced Renal-Cell Carcinoma**. *N Engl J Med* 2018, **378**(14):1277-1290.

33.	Mahoney KM, Ross-Macdonald P, Yuan L, Song L, Veras E, Wind-Rotolo M, McDermott DF, Stephen Hodi F, Choueiri TK, Freeman GJ: **Soluble PD-L1 as an early marker of progressive disease on nivolumab**. *J Immunother Cancer* 2022, **10**(2).

34.	Miao D, Margolis CA, Gao W, Voss MH, Li W, Martini DJ, Norton C, Bossé D, Wankowicz SM, Cullen D *et al*: **Genomic correlates of response to immune checkpoint therapies in clear cell renal cell carcinoma**. *Science* 2018, **359**(6377):801-806.

35.	Ascierto ML, McMiller TL, Berger AE, Danilova L, Anders RA, Netto GJ, Xu H, Pritchard TS, Fan J, Cheadle C *et al*: **The Intratumoral Balance between Metabolic and Immunologic Gene Expression Is Associated with Anti-PD-1 Response in Patients with Renal Cell Carcinoma**. *Cancer Immunol Res* 2016, **4**(9):726-733.

36.	Motzer RJ, Banchereau R, Hamidi H, Powles T, McDermott D, Atkins MB, Escudier B, Liu LF, Leng N, Abbas AR *et al*: **Molecular Subsets in Renal Cancer Determine Outcome to Checkpoint and Angiogenesis Blockade**. *Cancer Cell* 2020, **38**(6):803-817.e804.

37.	Motzer RJ, Robbins PB, Powles T, Albiges L, Haanen JB, Larkin J, Mu XJ, Ching KA, Uemura M, Pal SK *et al*: **Avelumab plus axitinib versus sunitinib in advanced renal cell carcinoma: biomarker analysis of the phase 3 JAVELIN Renal 101 trial**. *Nat Med* 2020, **26**(11):1733-1741.

38.	Liu S, Knochelmann HM, Lomeli SH, Hong A, Richardson M, Yang Z, Lim RJ, Wang Y, Dumitras C, Krysan K *et al*: **Response and recurrence correlates in individuals treated with neoadjuvant anti-PD-1 therapy for resectable oral cavity squamous cell carcinoma**. *Cell Rep Med* 2021, **2**(10):100411.

39.	Phillips D, Matusiak M, Gutierrez BR, Bhate SS, Barlow GL, Jiang S, Demeter J, Smythe KS, Pierce RH, Fling SP *et al*: **Immune cell topography predicts response to PD-1 blockade in cutaneous T cell lymphoma**. *Nat Commun* 2021, **12**(1):6726.

40.	van den Ende T, de Clercq NC, van Berge Henegouwen MI, Gisbertz SS, Geijsen ED, Verhoeven RHA, Meijer SL, Schokker S, Dings MPG, Bergman J *et al*: **Neoadjuvant Chemoradiotherapy Combined with Atezolizumab for Resectable Esophageal Adenocarcinoma: A Single-arm Phase II Feasibility Trial (PERFECT)**. *Clin Cancer Res* 2021, **27**(12):3351-3359.

41.	Zhao J, Chen AX, Gartrell RD, Silverman AM, Aparicio L, Chu T, Bordbar D, Shan D, Samanamud J, Mahajan A *et al*: **Immune and genomic correlates of response to anti-PD-1 immunotherapy in glioblastoma**. *Nat Med* 2019, **25**(3):462-469.

42.	Kim ST, Cristescu R, Bass AJ, Kim KM, Odegaard JI, Kim K, Liu XQ, Sher X, Jung H, Lee M *et al*: **Comprehensive molecular characterization of clinical responses to PD-1 inhibition in metastatic gastric cancer**. *Nat Med* 2018, **24**(9):1449-1458.

43.	Obradovic A, Graves D, Korrer M, Wang Y, Roy S, Naveed A, Xu Y, Luginbuhl A, Curry J, Gibson M *et al*: **Immunostimulatory Cancer-Associated Fibroblast Subpopulations Can Predict Immunotherapy Response in Head and Neck Cancer**. *Clin Cancer Res* 2022, **28**(10):2094-2109.

44.	Cho JW, Hong MH, Ha SJ, Kim YJ, Cho BC, Lee I, Kim HR: **Genome-wide identification of differentially methylated promoters and enhancers associated with response to anti-PD-1 therapy in non-small cell lung cancer**. *Exp Mol Med* 2020, **52**(9):1550-1563.

45.	Jung H, Kim HS, Kim JY, Sun JM, Ahn JS, Ahn MJ, Park K, Esteller M, Lee SH, Choi JK: **DNA methylation loss promotes immune evasion of tumours with high mutation and copy number load**. *Nat Commun* 2019, **10**(1):4278.

46.	Patil NS, Nabet BY, Müller S, Koeppen H, Zou W, Giltnane J, Au-Yeung A, Srivats S, Cheng JH, Takahashi C *et al*: **Intratumoral plasma cells predict outcomes to PD-L1 blockade in non-small cell lung cancer**. *Cancer Cell* 2022, **40**(3):289-300.e284.

47.	Auslander N, Zhang G, Lee JS, Frederick DT, Miao B, Moll T, Tian T, Wei Z, Madan S, Sullivan RJ *et al*: **Robust prediction of response to immune checkpoint blockade therapy in metastatic melanoma**. *Nat Med* 2018, **24**(10):1545-1549.

48.	Pomeranz Krummel DA, Nasti TH, Izar B, Press RH, Xu M, Lowder L, Kallay L, Rupji M, Rosen H, Su J *et al*: **Impact of Sequencing Radiation Therapy and Immune Checkpoint Inhibitors in the Treatment of Melanoma Brain Metastases**. *Int J Radiat Oncol Biol Phys* 2020, **108**(1):157-163.

49.	Hugo W, Zaretsky JM, Sun L, Song C, Moreno BH, Hu-Lieskovan S, Berent-Maoz B, Pang J, Chmielowski B, Cherry G *et al*: **Genomic and Transcriptomic Features of Response to Anti-PD-1 Therapy in Metastatic Melanoma**. *Cell* 2016, **165**(1):35-44.

50.	Riaz N, Havel JJ, Makarov V, Desrichard A, Urba WJ, Sims JS, Hodi FS, Martín-Algarra S, Mandal R, Sharfman WH *et al*: **Tumor and Microenvironment Evolution during Immunotherapy with Nivolumab**. *Cell* 2017, **171**(4):934-949.e916.

51.	Liu D, Schilling B, Liu D, Sucker A, Livingstone E, Jerby-Arnon L, Zimmer L, Gutzmer R, Satzger I, Loquai C *et al*: **Integrative molecular and clinical modeling of clinical outcomes to PD1 blockade in patients with metastatic melanoma**. *Nat Med* 2019, **25**(12):1916-1927.

52.	Gide TN, Quek C, Menzies AM, Tasker AT, Shang P, Holst J, Madore J, Lim SY, Velickovic R, Wongchenko M *et al*: **Distinct Immune Cell Populations Define Response to Anti-PD-1 Monotherapy and Anti-PD-1/Anti-CTLA-4 Combined Therapy**. *Cancer Cell* 2019, **35**(2):238-255.e236.

53.	Chiappinelli KB, Strissel PL, Desrichard A, Li H, Henke C, Akman B, Hein A, Rote NS, Cope LM, Snyder A *et al*: **Inhibiting DNA Methylation Causes an Interferon Response in Cancer via dsRNA Including Endogenous Retroviruses**. *Cell* 2017, **169**(2):361.

54.	Lauss M, Donia M, Harbst K, Andersen R, Mitra S, Rosengren F, Salim M, Vallon-Christersson J, Törngren T, Kvist A *et al*: **Mutational and putative neoantigen load predict clinical benefit of adoptive T cell therapy in melanoma**. *Nat Commun* 2017, **8**(1):1738.

55.	Garcia-Diaz A, Shin DS, Moreno BH, Saco J, Escuin-Ordinas H, Rodriguez GA, Zaretsky JM, Sun L, Hugo W, Wang X *et al*: **Interferon Receptor Signaling Pathways Regulating PD-L1 and PD-L2 Expression**. *Cell Rep* 2017, **19**(6):1189-1201.

56.	Kraehenbuehl L, Holland A, Armstrong E, O'Shea S, Mangarin L, Chekalil S, Johnston A, Bomalaski JS, Erinjeri JP, Barker CA *et al*: **Pilot Trial of Arginine Deprivation Plus Nivolumab and Ipilimumab in Patients with Metastatic Uveal Melanoma**. *Cancers (Basel)* 2022, **14**(11).

57.	Khalaf K, Hana D, Chou JT, Singh C, Mackiewicz A, Kaczmarek M: **Aspects of the Tumor Microenvironment Involved in Immune Resistance and Drug Resistance**. *Front Immunol* 2021, **12**:656364.

58.	He Y, Jiang Z, Chen C, Wang X: **Classification of triple-negative breast cancers based on Immunogenomic profiling**. *J Exp Clin Cancer Res* 2018, **37**(1):327.

59.	Bagaev A, Kotlov N, Nomie K, Svekolkin V, Gafurov A, Isaeva O, Osokin N, Kozlov I, Frenkel F, Gancharova O *et al*: **Conserved pan-cancer microenvironment subtypes predict response to immunotherapy**. *Cancer Cell* 2021, **39**(6):845-865.e847.

60.	Hänzelmann S, Castelo R, Guinney J: **GSVA: gene set variation analysis for microarray and RNA-seq data**. *BMC Bioinformatics* 2013, **14**:7.

61.	Wilkerson MD, Hayes DN: **ConsensusClusterPlus: a class discovery tool with confidence assessments and item tracking**. *Bioinformatics* 2010, **26**(12):1572-1573.

62.	Yoshihara K, Shahmoradgoli M, Martínez E, Vegesna R, Kim H, Torres-Garcia W, Treviño V, Shen H, Laird PW, Levine DA *et al*: **Inferring tumour purity and stromal and immune cell admixture from expression data**. *Nat Commun* 2013, **4**:2612.

63.	Brunet JP, Tamayo P, Golub TR, Mesirov JP: **Metagenes and molecular pattern discovery using matrix factorization**. *Proc Natl Acad Sci U S A* 2004, **101**(12):4164-4169.

64.	Yu G, Wang LG, Han Y, He QY: **clusterProfiler: an R package for comparing biological themes among gene clusters**. *Omics* 2012, **16**(5):284-287.

65.	Ben-Salem S, Venkadakrishnan VB, Heemers HV: **Novel insights in cell cycle dysregulation during prostate cancer progression**. *Endocr Relat Cancer* 2021, **28**(6):R141-r155.

66.	Mantovani A, Dinarello CA, Molgora M, Garlanda C: **Interleukin-1 and Related Cytokines in the Regulation of Inflammation and Immunity**. *Immunity* 2019, **50**(4):778-795.

67.	Hoshida Y, Brunet JP, Tamayo P, Golub TR, Mesirov JP: **Subclass mapping: identifying common subtypes in independent disease data sets**. *PLoS One* 2007, **2**(11):e1195.

68.	Zheng K, Gao L, Hao J, Zou X, Hu X: **An immunotherapy response prediction model derived from proliferative CD4(+) T cells and antigen-presenting monocytes in ccRCC**. *Front Immunol* 2022, **13**:972227.

69.	Au L, Hatipoglu E, Robert de Massy M, Litchfield K, Beattie G, Rowan A, Schnidrig D, Thompson R, Byrne F, Horswell S *et al*: **Determinants of anti-PD-1 response and resistance in clear cell renal cell carcinoma**. *Cancer Cell* 2021, **39**(11):1497-1518.e1411.

70.	Athar A, Füllgrabe A, George N, Iqbal H, Huerta L, Ali A, Snow C, Fonseca NA, Petryszak R, Papatheodorou I *et al*: **ArrayExpress update - from bulk to single-cell expression data**. *Nucleic Acids Res* 2019, **47**(D1):D711-d715.

71.	Gulati GS, Sikandar SS, Wesche DJ, Manjunath A, Bharadwaj A, Berger MJ, Ilagan F, Kuo AH, Hsieh RW, Cai S *et al*: **Single-cell transcriptional diversity is a hallmark of developmental potential**. *Science* 2020, **367**(6476):405-411.

72.	Malta TM, Sokolov A, Gentles AJ, Burzykowski T, Poisson L, Weinstein JN, Kamińska B, Huelsken J, Omberg L, Gevaert O *et al*: **Machine Learning Identifies Stemness Features Associated with Oncogenic Dedifferentiation**. *Cell* 2018, **173**(2):338-354.e315.

73.	Subramanian A, Tamayo P, Mootha VK, Mukherjee S, Ebert BL, Gillette MA, Paulovich A, Pomeroy SL, Golub TR, Lander ES *et al*: **Gene set enrichment analysis: a knowledge-based approach for interpreting genome-wide expression profiles**. *Proc Natl Acad Sci U S A* 2005, **102**(43):15545-15550.

74.	Langfelder P, Horvath S: **WGCNA: an R package for weighted correlation network analysis**. *BMC Bioinformatics* 2008, **9**:559.

75.	Hao Y, Hao S, Andersen-Nissen E, Mauck WM, 3rd, Zheng S, Butler A, Lee MJ, Wilk AJ, Darby C, Zager M *et al*: **Integrated analysis of multimodal single-cell data**. *Cell* 2021, **184**(13):3573-3587.e3529.

76.	Aran D, Looney AP, Liu L, Wu E, Fong V, Hsu A, Chak S, Naikawadi RP, Wolters PJ, Abate AR *et al*: **Reference-based analysis of lung single-cell sequencing reveals a transitional profibrotic macrophage**. *Nat Immunol* 2019, **20**(2):163-172.

77.	Zou J, Deng F, Wang M, Zhang Z, Liu Z, Zhang X, Hua R, Chen K, Zou X, Hao J: **scCODE: an R package for data-specific differentially expressed gene detection on single-cell RNA-sequencing data**. *Brief Bioinform* 2022, **23**(5).

78.	Ritchie ME, Phipson B, Wu D, Hu Y, Law CW, Shi W, Smyth GK: **limma powers differential expression analyses for RNA-sequencing and microarray studies**. *Nucleic Acids Res* 2015, **43**(7):e47.

79.	Șenbabaoğlu Y, Michailidis G, Li JZ: **Critical limitations of consensus clustering in class discovery**. *Sci Rep* 2014, **4**:6207.

80.	Szklarczyk D, Kirsch R, Koutrouli M, Nastou K, Mehryary F, Hachilif R, Gable AL, Fang T, Doncheva NT, Pyysalo S *et al*: **The STRING database in 2023: protein-protein association networks and functional enrichment analyses for any sequenced genome of interest**. *Nucleic Acids Res* 2023, **51**(D1):D638-d646.

81.	Mayakonda A, Lin DC, Assenov Y, Plass C, Koeffler HP: **Maftools: efficient and comprehensive analysis of somatic variants in cancer**. *Genome Res* 2018, **28**(11):1747-1756.

82.	Zhang H, Meltzer P, Davis S: **RCircos: an R package for Circos 2D track plots**. *BMC Bioinformatics* 2013, **14**:244.

83.	Newman AM, Liu CL, Green MR, Gentles AJ, Feng W, Xu Y, Hoang CD, Diehn M, Alizadeh AA: **Robust enumeration of cell subsets from tissue expression profiles**. *Nat Methods* 2015, **12**(5):453-457.

84.	Fu J, Li K, Zhang W, Wan C, Zhang J, Jiang P, Liu XS: **Large-scale public data reuse to model immunotherapy response and resistance**. *Genome Med* 2020, **12**(1):21.

85.	Yang W, Soares J, Greninger P, Edelman EJ, Lightfoot H, Forbes S, Bindal N, Beare D, Smith JA, Thompson IR *et al*: **Genomics of Drug Sensitivity in Cancer (GDSC): a resource for therapeutic biomarker discovery in cancer cells**. *Nucleic Acids Res* 2013, **41**(Database issue):D955-961.

86.	Rees MG, Seashore-Ludlow B, Cheah JH, Adams DJ, Price EV, Gill S, Javaid S, Coletti ME, Jones VL, Bodycombe NE *et al*: **Correlating chemical sensitivity and basal gene expression reveals mechanism of action**. *Nat Chem Biol* 2016, **12**(2):109-116.

87.	Maeser D, Gruener RF, Huang RS: **oncoPredict: an R package for predicting in vivo or cancer patient drug response and biomarkers from cell line screening data**. *Brief Bioinform* 2021, **22**(6).
